# Extreme Osmotolerance and Halotolerance in Food-Relevant Yeasts and the Role of Glycerol-Dependent Cell Individuality

**DOI:** 10.3389/fmicb.2018.03238

**Published:** 2019-01-09

**Authors:** Malcolm Stratford, Hazel Steels, Michaela Novodvorska, David B. Archer, Simon V. Avery

**Affiliations:** School of Life Sciences, University of Nottingham, Nottingham, United Kingdom

**Keywords:** population diversity, Tps1, resistant sub-population, HOG pathway bet-hedging, spoilage

## Abstract

Osmotolerance or halotolerance are used to describe resistance to sugars and salt, or only salt, respectively. Here, a comprehensive screen of more than 600 different yeast isolates revealed that osmosensitive species were equally affected by NaCl and glucose. However, the relative toxicity of salt became increasingly prominent in more osmoresistant species. We confirmed that growth inhibition by glucose in a laboratory strain of *Saccharomyces cerevisiae* occurred at a lower water activity (A_w_) than by salt (NaCl), and pre-growth in high levels of glucose or salt gave enhanced cross-resistance to either. Salt toxicity was largely due to osmotic stress but with an additive enhancement due to effects of the relevant cation. Almost all of the yeast isolates from the screen were also noted to exhibit hetero-resistance to both salt and sugar, whereby high concentrations restricted growth to a small minority of cells within the clonal populations. Rare resistant colonies required growth for up to 28 days to become visible. This cell individuality was more marked with salt than sugar, a possible further reflection of the ion toxicity effect. In both cases, heteroresistance in *S. cerevisiae* was strikingly dependent on the *GPD1* gene product, important for glycerol synthesis. In contrast, a *tps1Δ* deletant impaired for trehalose showed altered MIC but no change in heteroresistance. Effects on heteroresistance were evident in chronic (but not acute) salt or glucose stress, particularly relevant to growth on low A_w_ foods. The study reports diverse osmotolerance and halotolerance phenotypes and heteroresistance across an extensive panel of yeast isolates, and indicates that Gpd1-dependent glycerol synthesis is a key determinant enabling growth of rare yeast subpopulations at low A_w_, brought about by glucose and in particular salt.

## Introduction

Microorganisms are subject to osmotic stress in their natural environments and in industrial settings, while their osmotolerance can be manipulated to control or enhance microbial growth (Lievens et al., 2015; Vyrides and Stuckey, 2017). Consequently, the physiological and molecular bases of microbial osmotolerance and responses to osmotic stress are very well studied, which has also helped shape our understanding of corresponding responses in higher organisms (Hohmann, 2002; Smith et al., 2010). As well as osmotic stress encountered in the natural environment, preservation of foods by dehydration is one of the most traditional methods for preventing spoilage by yeasts and molds and growth of food-poisoning bacteria. Besides the drying of foods, dehydration is achieved by addition of salts or sugars, resulting in osmotic stress to microbes in contact with the food, e.g., preserves, fruit sauces, salted meat, etc. The dehydration level of different foods is typically evaluated by the water activity (A_w_), which indicates the concentration of freely mobile water molecules (Russell and Gould, 2003). This is normally measured by the water vapor pressure, in equilibrium at a set temperature above the food sample.

Dehydration with solutes such as salts or sugars can be due to different properties of the solutes. Sugar solutions cause reductions in water activity largely by physical displacement of water molecules, whereas salt solutions lower water activity largely by binding water molecules (Kasaai, 2014). One liter of pure water (100% w/v) contains 55.55 M water, whereas 1 l of 50% (w/v) sugar syrup contains far less than a liter of water due to the physical displacement by the sugar volume. As a result, measurements of water activity are commonly referred to as percentage weight/weight (% w/w), rather than % w/v.

Fortunately, most bacteria, including food-poisoning bacteria such as *Salmonella* spp. and *Clostridium botulinum*, are sensitive to osmotic stress and few will proliferate in foods with an A_w_ below 0.92. *Staphylococcus aureus* is the most osmotolerant pathogen, growing down to A_w_ 0.86 if oxygen is present (Russell and Gould, 2003; Grant, 2004). Many yeast and mold species are more osmotolerant and can grow at A_w_ 0.80 (e.g., relevant to fruit juice concentrates, syrups, condensed milk), while osmophilic molds and yeasts such as *Aspergillus* spp. and *Zygosaccharomyces rouxii* can grow slowly at A_w_ ∼0.61 (Dakal et al., 2014).

The sudden arrival of microorganisms into low water-activity environments, a rare event in the natural environment (Welsh, 2000), causes acute stress due to rapid dehydration. Yeast cells lose viability under high osmotic stress, in an apoptosis-like manner, and subsequent growth at low water activity is extremely slow (Tokuoka, 1993; Huh et al., 2002; Silva et al., 2005). Water molecules are normally in equilibrium across microbial plasma membranes, via diffusion through aquaporin channels (Gena et al., 2011). A sudden reduction in the external water concentration causes a drop in water influx while efflux continues, resulting in contraction of the plasma membrane and its cytoplasmic content. Osmotic stress in yeast causes shrinkage of both the membrane and the cell wall within a few seconds (Morris et al., 1986; Hohmann et al., 2007; Dupont et al., 2011). Membrane fluidity and composition and cell wall elasticity influences osmo-sensitivity and are altered in response to osmotic stress (Hosono, 1992; Rodriguez-Vargas et al., 2007; Ene et al., 2015). Membrane shrinkage in *Saccharomyces cerevisiae* triggers induction of the HOG (High Osmolarity Glycerol) signaling pathway (Hohmann, 2002; Talemi et al., 2016). The mitogen-activated protein kinase Hog1 translocates to the nucleus during osmotic stress and upregulates expression of a large number of genes, many involved in synthesis of the compatible solute glycerol (Pascual-Ahuir et al., 2018). Glycerol, which replaces water and increases osmotic turgor pressure within the cytoplasm, is accumulated due both to synthesis via glycerol 3-phosphate dehydrogenases encoded by *GPD1* and *GPD2* (Ansell et al., 1997), active uptake from the medium (Ferreira et al., 2005; Petelenz-Kurdziel et al., 2013), and prevention of leakage via the Fsp1p channel (Tamas et al., 1999; Sabir et al., 2017). Glycerol is the most common compatible solute in yeasts grown on glucose (Babazadeh et al., 2017), but other species accumulate arabitol or mannitol (Van Eck et al., 1993), or glycine/betaine in bacteria (Welsh, 2000). Nevertheless, metagenomic profiling of microbial populations from saline environments has shown enrichment of conserved osmotolerance gene functions (Ahmed et al., 2018).

It was reported that osmotic stress in bacterial cultures could be caused equally by a variety of solutes (Russell et al., 2003). However, yeasts isolated from natural salty environments are not those typically found growing in high sugar conditions (Butinar et al., 2005) and isolates from salty environments have different responses or tolerances to salt versus sugar (Solieri et al., 2014; Fredsgaard et al., 2017). This highlights a distinction between resistance to salt and sugar, i.e., halotolerance versus osmotolerance. Sodium ions from salt can penetrate the cell via potassium transporters, but are actively extruded via the Ena1 P-type ATPase and Nha1 antiporter systems (Arino et al., 2010; Dakal et al., 2014). Impairment of these systems results in salt sensitivity. Salt stress can inhibit activity of certain cellular enzymes (Glaser et al., 1993). Yet salt also exerts an osmotic pressure and many reports on the HOG pathway were carried out using moderate salt (NaCl) concentrations. Thus, although there is accepted wisdom on the similarities in cellular responses to osmo- and halo-tolerance, there is an important distinction. A main initial aim of this study was to interrogate that distinction across a resource of more than 600 food-relevant yeast isolates.

Previous work had shown than the resistance of yeasts to preservatives or other inhibitors is subject to population heterogeneity (Steels et al., 2000; Holland et al., 2014; Stratford et al., 2014; Altamirano et al., 2018): despite genetic uniformity of the cell population, the bulk population was only moderately resistant and a very small sub-population showed high resistance, i.e., heteroresistance. This indicated that population growth (e.g., associated with spoilage) was largely due to the presence of rare, resistant individuals within the population.

In this paper, we resolved halo- and osmo-tolerance across a wide diversity of yeast isolates and related such phenotypes to the prevalence of heteroresistance and its basis, which could have important consequences for microbial proliferation in high salt and high sugar conditions.

## Materials and Methods

### Yeast Strains

A total of 626 yeast strains were used in this study, listed in Supplementary Table S1 together with their sources of isolation. The identity of all strains was confirmed by sequencing the D1/D2 region of the 26S rDNA using previously described methods (Kurtzman, 2003). In addition, *S. cerevisiae* BY4741 and isogenic deletions strains were obtained from Euroscarf (Frankfurt): *gpd1Δ* (YDL022W) affecting glycerol synthesis via glycerol-3-phosphate dehydrogenase, and *tps1Δ* (YBR126C) affecting trehalose synthesis and energy homeostasis regulation via trehalose-6-phosphate synthase (Petitjean et al., 2015). Strains were stored in glycerol on ceramic beads at -80°C (Microbank^TM^), and maintained short term on MEA (malt extract agar, Oxoid) slopes at 4°C.

### Growth Media and Conditions

The growth medium used in this study was YEPD, containing glucose 20 g/l (0.11 M), bacteriological peptone (Oxoid) 20 g/l, and yeast extract (Oxoid) 10 g/l, adjusted to pH 4.0 with 5 M HCl prior to heat sterilization. Yeast starter cultures comprised 10 ml YEPD pH 4.0 in 28 ml McCartney bottles, inoculated with yeast from MEA slopes, and incubated to stationary phase at 48 h at 25°C. Experimental cultures were inoculated with a population of 10^4^ cells (10^1^ cells/ml) of yeast cells into 10 ml aliquots of YEPD pH 4.0 in 28 ml McCartney bottles.

### Measurement of Resistance to Salt and Glucose (MIC Tests)

Resistance of yeast strains to salt and sugar in broth cultures was investigated by determination of the minimum inhibitory concentration (MIC) to completely inhibit growth. Series of McCartney bottles were prepared with 10 ml aliquots of YEPD, each containing a progressively higher concentration of salts or sugars. The pH of all media was back titrated to pH 4.0 following salt/sugar additions. Typically, a large volume of a high concentration was prepared, e.g., 4 M NaCl, and the pH of the YEPD was corrected to pH 4.0. This was then mixed with YEPD pH 4.0 in different proportions to yield a range of concentrations, all at pH 4.0. After heat treatment, bottles were inoculated with 10^3^ cells/ml and incubated statically for 28 days at 25°C. The MIC was the lowest concentration of preservative at which no growth was detectable at 28 days. FIC (fractional inhibitory concentration) index was determined as described previously (Vallieres et al., 2018). In a previous study it was found that in static cultures like these, yeast cells slowly sink through liquid and form distinct, countable colonies in McCartney bottles and in Microtiter dishes (Stratford et al., 2013). In the current study, such discrete colonies were countable after 28 days in McCartney bottles containing media close to the MIC level of salt; similar tests could not be carried out using glucose because most yeast cells floated in high-density glucose (above 2 M) and did not form colonies.

### Measurement of Water Activity (A_w_)

The water activity of media was directly measured using an Aqualab 4TEV water activity meter^2^ supplied by Labcell Ltd., Alton, Hants, United Kingdom, GU34 5PZ^3^. The water activity level was determined in each case in YEPD media, and calibration curves were prepared for the salt and glucose molarities against A_w_ in the same medium. The meter was maintained at 25°C, and 5 ml samples were pre-incubated for 30 min at 25°C, before initiating measurement.

### Osmotic Shock Death Curve Measurement and Heteroresistance

Time-dependent osmotic death curves (acute osmotic stress) were typically initiated by inoculation of 10^4^ *S. cerevisiae* cells into 10 ml YEPD pH 4.0, or pH 6.0, containing either 2 M salt (NaCl) or 3 M glucose at 25°C. Survival was assessed at indicated time points by spread plating 100 μl samples onto YEPD agar and incubation at 25°C for 5–7 days to allow colony formation. Due to the possible osmotic down-shock onto the agar, tests were carried out using high glucose YEPD agar (1 M) but the results showed that the yeast counts were nearly identical to those on normal YEPD agar.

In the case of resistance to chronic osmotic stress, yeast cells from cultures cultivated in YEPD broth were spread plated to agar supplemented with indicated concentrations of NaCl or glucose. Plates were incubated at 25°C before assessment of colony forming ability.

Heteroresistance was determined according to the gradients of plots of % colony formation against concentration of NaCl or glucose; shallower slopes indicate greater heterogeneity (Holland et al., 2014; Hewitt et al., 2016). For quantitative comparison, Hill slopes were fitted to plots (% Viability vs. log_10_[Salt/Sugar]) using Prism software and arctangent values for the slopes calculated with Excel to estimate relative heterogeneity.

### Determination of Population Heterogeneity According to the Inoculum Effect

To measure the proportion of a yeast cell population capable of growth in increasing osmotic concentrations, stationary phase *S. cerevisiae* cells from 2-day starter cultures were spread onto YEPD agar plates at 10^4^, 10^3^, and 10^2^ cells per plate. Osmotic concentrations ranged from 0–2.4 M NaCl and 0–3.4 M glucose, and the agar pH was corrected to pH 6.0. Due to the effect of salt elevating agar melting temperatures, agars were cooled to 60°C after autoclaving, before pouring into Petri dishes. Samples were incubated at 25°C for 28 days with colony counting every 2–3 days.

### Measurement of Glycerol and Trehalose Content

Yeast cells were grown in 10 ml static cultures of YEPD and the cell numbers counted by haemocytometer after 2 weeks. Cells were broken open using a Mikro-Dismembrator U instrument (Sartorius Stedim Biotech, Germany) for 2 min at a shaking frequency of 2000/min. D-trehalose content was analyzed using a commercial kit (Megazyme International Ireland Ltd.) using the specified protocol^3^. Glycerol was analyzed by HPLC (Agilent Technologies 1200 series) using a 7.8 mm × 300 mm ion exclusion column (Aminex HPX-87H, Bio-Rad Laboratories Inc., Hertfordshire, United Kingdom). Detection was carried out using a refractive index detector and Agilent Chemstation software, version B.04.02.

## Results

### Salt and Sugar Toxicity Against Diverse Yeast Strains and Species

Inhibition by salt and glucose was compared in a total of 626 yeast strains within 151 species growing in YEPD pH 4.0 (Supplementary Table S1). Overall, the yeast species tended to be more resistant to glucose than salt (mean MIC glucose, 3.208 M; mean MIC salt, 2.316 M). A summary for different yeast species is shown in Table 1. The most glucose-resistant species tested were *Zygosaccharomyces* spp. while salt resistant species included *Debaryomyces hansenii* var. *fabryii* as well as *Candida parapsilosis*. Other yeast species noted here as salt sensitive, as compared with their glucose resistance, included *Candida apicola, Torulaspora microellipsoides* and *Zygotorulaspora florentina*, and certain individual strains within *Candida pseudointermedia, Clavispora lusitania, Wickerhamomyces anomalus* and *Zygosaccharomyces bailii*. Across ranges of strains, this new dataset for yeasts supports the widely recognized sugar resistance of *Z. rouxii*, salt resistance of *D. hansenii* and salt sensitivity of *S. pombe*, and reveal similar differences in a range of other species.

**Table 1:** Osmotolerance and halotolerance of yeast species.

Number of strains	Yeast species	MIC Glucose (M)	MIC NaCl (M)	Ratio
2	*Candida albicans*	3.68 ± 0.53	2.53 ± 0.67	1.455
1	*Candida apicola*	4.50	1.40	3.214
23	*Candida parapsilosis*	3.54 ± 0.34	3.24 ± 0.23	1.093
33	*Candida pseudointermedia*	2.96 ± 0.15	2.21 ± 0.37	1.344
19	*Clavispora lusitaniae*	3.12 ± 0.29	2.35 ± 0.35	1.326
12	*Cryptococcus laurentii*	2.93 ± 0.18	2.11 ± 0.32	1.385
5	*Cryptococcus liquefaciens*	2.27 ± 0.14	2.17 ± 0.30	1.046
6	*Debaryomyces hansenii*	3.78 ± 0.39	2.84 ± 0.60	1.331
2	*Debaryomyces hansenii* var. *fabryii*	3.93 ± 0.11	3.35 ± 0.07	1.172
3	*Dekkera anomala*	1.88 ± 0.33	1.32 ± 0.08	1.430
8	*Dekkera bruxellensis*	1.93 ± 0.24	1.25 ± 0.18	1.545
3	*Kazachstania exigua*	2.92 ± 0.14	1.97 ± 0.15	1.483
12	*Meyerozyma guilliermondii*	3.34 ± 0.19	2.82 ± 0.54	1.186
2	*Pichia membranifaciens*	2.83 ± 0.11	2.55 ± 0.92	1.108
18	*Rhodotorula mucilaginosa*	2.88 ± 0.29	2.48 ± 0.19	1.162
6	*Saccharomyces bayanus*	2.95 ± 0.12	2.06 ± 0.21	1.433
32	*Saccharomyces cerevisiae*	3.21 ± 0.29	2.04 ± 0.21	1.577
2	*Schizosaccharomyces pombe*	3.88 ± 0.53	0.85 ± 0.35	4.559
10	*Torulaspora delbrueckii*	3.64 ± 0.17	2.66 ± 0.11	1.369
3	*Torulaspora microellipsoides*	3.02 ± 0.03	0.75 ± 0.09	4.022
17	*Wickerhamomyces anomalus*	3.59 ± 0.22	2.65 ± 0.40	1.359
10	*Yarrowia lipolytica*	2.88 ± 0.17	2.45 ± 0.13	1.176
44	*Zygosaccharomyces bailii*	3.96 ± 0.21	2.70 ± 0.35	1.464
15	*Zygosaccharomyces bisporus*	4.05 ± 0.40	2.78 ± 0.30	1.455
12	*Zygosaccharomyces lentus*	3.67 ± 0.18	2.35 ± 0.24	1.557
9	*Zygosaccharomyces mellis*	4.86 ± 0.15	3.12 ± 0.39	1.557
29	*Zygosaccharomyces rouxii*	4.89 ± 0.53	3.43 ± 0.32	1.425
2	*Zygotorulaspora florentina*	3.55 ± 0.07	1.55 ± 0.07	2.290

*Strains were inoculated (10^4^ cells) into 10 ml YEPD pH 4.0 – containing increasing concentrations of salt or glucose to determine the minimum inhibitory concentration (MIC) after incubation at 25°C for 2 weeks. The full data are listed in Supplementary Table S1.*

**FIGURE 1:**
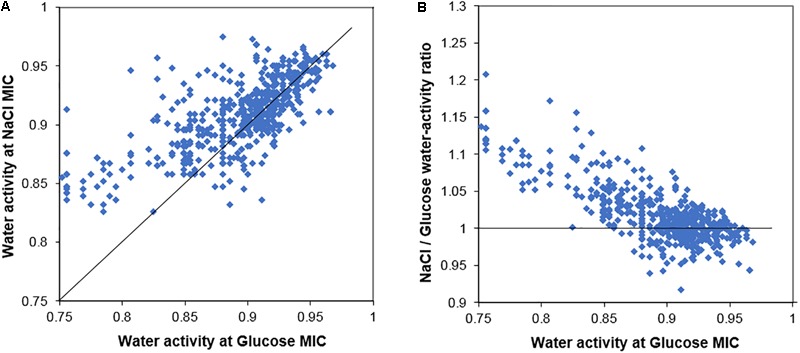
Comparison of the salt and glucose resistance of 626 yeast strains. **(A)** Scatter plot of the MICs of salt (NaCl) and glucose for yeast strains, measured in terms of water activity, A_w_. **(B)** Ratio of salt/glucose MIC (A_w_) scatter plotted against the glucose MIC (A_w_); the ratio becomes notably higher (relative sensitization to salt) in yeasts with high resistance to glucose. The 45° line **(A)** and line showing ratio value = 1 **(B)** are described in the main text.

In general terms, strains of yeasts from the screen that were resistant to glucose toxicity were also resistant to salt toxicity. Figure 1A presents a scatter plot of the MIC salt against the MIC glucose for the 626 yeast strains, measured in terms of water activity (A_w_). If salt and glucose both acted only by osmotic stress, the data would follow a 45° line of equal A_w_, a 1:1 ratio. The data instead contain a large number of strains with points above the 45° line showing higher sensitivity to salt after accounting for the effect of A_w_. A further scatter (Eadie/Hofstee type) plot shows the ratio of salt/glucose MIC A_w_ for each strain plotted against the glucose A_w_ (Figure 1B). If both salt and glucose acted by osmotic stress only, all the data should follow a horizontal line with ratio value ∼1. Figure 1B shows that this is largely so for osmo-sensitive species, growing in glucose ranging from A_w_ 1.00 to 0.90. However, for more osmoresistant species, the scatter shows the plots rising with a higher ratio, indicating that these strains are showing a progressive sensitivity to salt. The data demonstrate that osmosensitive strains do not show evidence of salt toxicity, but as strains become increasingly resistant to osmotic stress, the salt toxicity becomes increasingly apparent.

### Comparison of Salt and Sugar Toxicity in *Saccharomyces cerevisiae*

Additional tests were performed with the model yeast *S. cerevisiae*, to help rationalize certain of the above observations. Preliminary data showed that *S. cerevisiae* BY4741 grew in YEPD medium, pH 4.0, up to an MIC of salt (NaCl) close to 2 M and an MIC of glucose close to 3 M. This represents a salt concentration of 11.7% w/v (11.1% w/w) and glucose concentration of 54% w/v (46.2% w/w). Note that the 2% w/v glucose concentration in standard YEPD already exceeds yeast needs for carbon source supply, meaning that further increases in glucose concentration allow osmotic stress to be exerted without a simultaneous effect on adequacy of carbon supply. The water activity of these MIC solutions was directly measured in YEPD: 2 M NaCl gave A_w_ 0.921, and 3 M glucose gave A_w_ 0.906, corroborating that the inhibitory concentrations of these compounds did not cause identical osmotic stress. To gain a similar A_w_ level, the salt concentration would need to be raised to ∼2.3–2.4 M. Therefore, the salt solution would be marginally more toxic than glucose to *S. cerevisiae* at the same A_w_.

**FIGURE 2:**
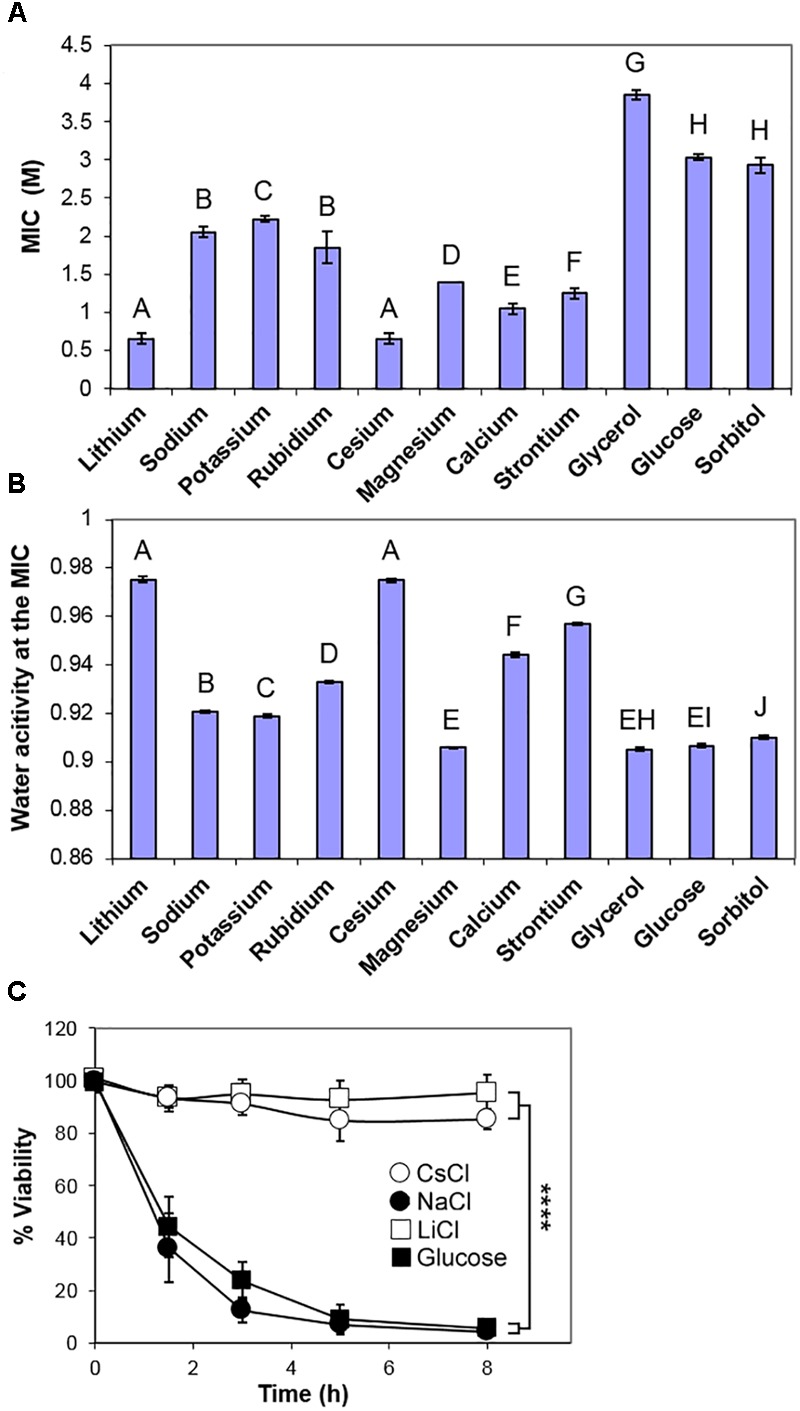
Comparison of inhibition of *S. cerevisiae* BY4741 by chloride salts, glucose and polyols. **(A)** Molar MICs of the indicated chloride salts, glycerol, glucose, and sorbitol, determined after 4 weeks incubation at 25°C in YEPD broth, pH 4.0. Data are means ± SD from three replicate determinations. Means with different letters are significantly different (unpaired *t*-test, *p* < 0.05). **(B)** Water activity A_w_ measurements of the mean MICs in YEPD, pH 4.0. **(C)** Death curves following inoculation into YEPD containing the MICs of sodium, lithium and cesium chlorides, and glucose. Samples were taken over time and plated out onto YEPD agar, before assessment of colony forming ability. Data are means ± SD from three replicate determinations. ^∗∗∗∗^*p* < 0.0001, unpaired *t-*test.

Other solutes tested in this study also required concentrations in excess of 0.5 M to inhibit growth of *S. cerevisiae*, but inhibitory concentrations (MICs) of a panel of periodic table Group 1 and Group 2 chloride salts varied by at least threefold (Figure 2A). Measurements of the osmotic stress (A_w_) at each of the MIC concentrations showed a consistent low water activity (0.905–0.910) for glucose, glycerol, sorbitol, and magnesium chloride (Figure 2B) as well as other tested sugars (fructose, mannose, and galactose; data not shown). Sodium and potassium chlorides inhibited at a marginally higher A_w_ (Figure 2B) while lithium and cesium chlorides inhibited at much lower concentrations, with a high A_w_ close to 0.975. The relative toxicities of the different salts supported conclusions from previous studies (Petrezselyova et al., 2010). The apparent similarity of NaCl and KCl toxicities (Figure 2B) should be weighed against the fact that much less Na^+^ than K^+^ is normally accumulated by cells; it does not suggest that intracellular K^+^ is near equi-toxic with intracellular Na^+^. The data are consistent with: sugars (glucose, mannose, fructose, galactose), polyols (glycerol, sorbitol) and magnesium chloride inhibiting *S. cerevisiae* by osmotic stress only; potassium and sodium chlorides acting primarily by osmotic stress, but with a contribution from Na^+^ and K^+^ cation toxicity; rubidium, calcium and strontium chlorides showing further relative weightings toward cation toxicity; LiCl and CsCl acting primarily by the toxicity of the individual ions, rather than by osmotic stress.

One characteristic of osmotic stress is to cause a high proportion of a yeast population to undergo apoptosis-like cell death over 4–8 h following immersion at low A_w_ (Huh et al., 2002; Granot et al., 2003). Therefore, different solutes of this study were compared by inoculation of *S. cerevisiae* BY4741 into the inhibitory concentration of each (MIC), before plating onto standard solid medium to assess viability (colony forming ability). Note that this short-term, loss-of-viability assay is a measure of resistance to acute stress, whereas the preceding tests above, of long-term growth in the presence of stressor, related more to chronic resistance; this distinction is addressed further below. The results corroborated that both salt (NaCl) and sugar (glucose) cause marked cell death (>90%) within 8 h. At the MIC levels of LiCl and CsCl, significant cell death was not detected over the same timeframe (Figure 2C) showing that cells could retain viability in the presence of these ions and recommence growth when the solutes were removed. Other osmotic stress solutes, glycerol, sorbitol, magnesium chloride, caused very substantial cell death. However, the more intermediate A_w_ solutes, such as calcium, strontium and rubidium chlorides, caused a slower cell death (Supplementary Figure S1).

### Additive Effect of Salt and Sugar Toxicity Against *S. cerevisiae*

**FIGURE 3:**
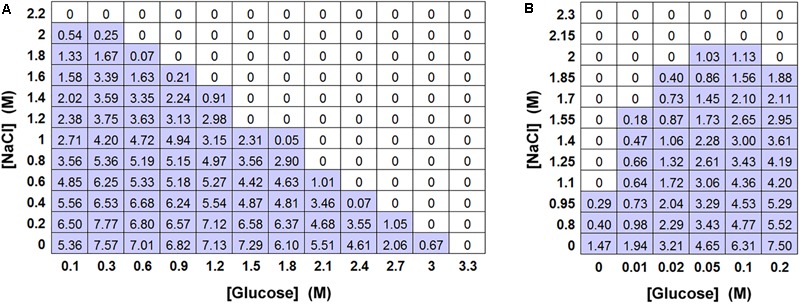
Effect of combinations of salt and glucose in inhibiting *S. cerevisiae* BY4741. **(A)** Matrix-type experiment of combinations of salt (NaCl) and glucose in inhibiting *S. cerevisiae* BY4741 (10^4^ cells), in 10 ml aliquots of YEPD pH 4.0 after incubation at 25°C for 4 weeks. All values represent the mean OD_600_ from triplicate determinations (SDs averaged <10% of the mean values shown). **(B)** The effect of low glucose levels on resistance to NaCl in a matrix-type experiment, conditions as described for **(A)**.

As the screen of yeast isolates showed that the relative toxicity of salt (NaCl) became increasingly prominent in more osmoresistant species (Table 1 and Figure 1B), a matrix-type experiment was set up to examine the effects of different combinations of salt and glucose in inhibiting the growth of *S. cerevisiae*. The results confirmed that the MICs of salt and glucose against *S. cerevisiae* were close to 2 M (A_w_ 0.921) and 3 M (A_w_ 0.906), respectively. They also showed that combinations of salt and glucose concentrations were essentially additive (Figure 3A), reflected by an FIC (fractional inhibitory concentration) index ∼1.0. For example, at half the MIC level of salt (1 M), half of the MIC level of glucose (1.5 M) was sufficient to approach the yeast inhibition level. Alternatively, 75% of the salt MIC could be combined with 25% of the glucose MIC and *vice versa*. It was noted that, whereas the increase in salt concentration up to the MIC progressively reduced the yeast growth yield (OD_600_), increasing the glucose concentration had little effect until fairly close to the MIC. Combinations of salt and glucose generally showed reduced growth in proportion to the salt concentration, irrespective of the glucose addition. This is an indication that the reduction in growth is due to salt toxicity, not to osmotic stress. Moreover, low glucose enhanced resistance to salt toxicity, with the salt MIC falling by 50% in the absence of glucose in YEPD (Figure 3B). Further glucose additions to concentrations >0.05 M additively made the yeast more sensitive again to salt toxicity.

### Population Heterogeneity in Resistance to Salt and Sugar Toxicity

**FIGURE 4:**
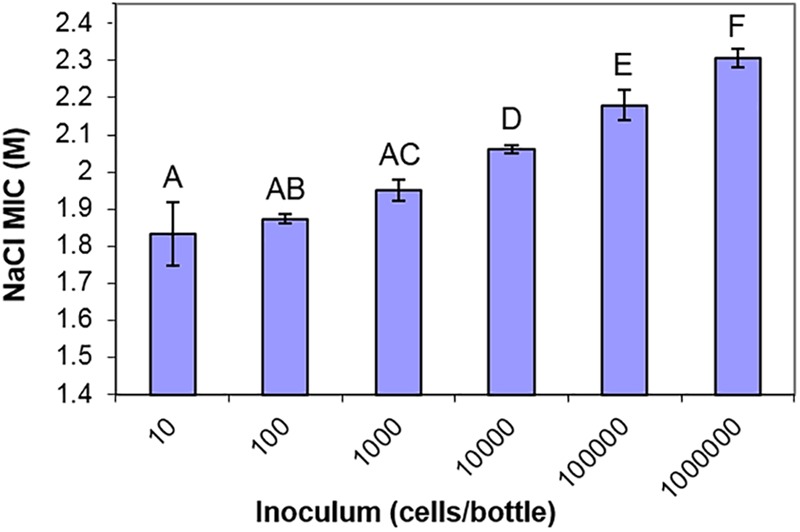
The inoculum effect of salt resistance. MIC values of *S. cerevisiae* BY4741 to NaCl determined from different starting inocula in 10 ml aliquots of YEPD pH 4.0, after incubation at 25°C for 4 weeks. Data are mean values from triplicate cultures ± SD. Means with different letters are significantly different (unpaired *t-*test, *p* < 0.05).

One further observation warranting closer investigation from the screen of yeast isolates was that, in the static cultures containing high concentrations of salt, very small numbers of yeast colonies were visible growing in the bottles. For almost all of the yeast strains, fewer than 10 colonies were visible in cultures close to the MIC of salt (Supplementary Figure S2). Since the yeast inoculum was 10^4^ cells/bottle, this indicated that only a small proportion of the cell population could grow in high salt, a reflection of population heterogeneity in salt resistance. Population heterogeneity has been widely reported in diverse single-celled organisms and phenotypes in recent years. Similar observations could not be performed in high glucose concentrations since most yeasts, being of lower density, do not sink in high glucose (above 2 M) and do not form distinct colonies. Prompted by this observation, we used *S. cerevisiae* to demonstrate an inoculum effect (Steels et al., 2000). The recorded resistance (MIC) progressively increased as the level of population inoculum was elevated (Figure 4). This indicated that a large cell population is required to contain a detectable sub-population of a few, rare salt-resistant cells.

**FIGURE 5:**
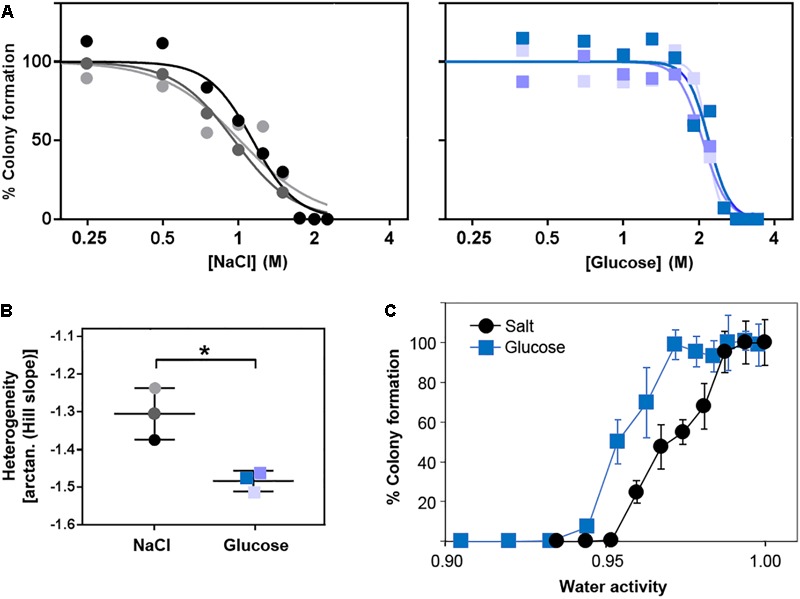
Heteroresistance of *S. cerevisiae* to chronic glucose or salt (NaCl) stress. *S. cerevisiae* BY4741 was cultured in YEPD broth then plated to YEPD supplemented with the indicated concentrations of glucose or NaCl. Colonies were enumerated after incubation for 28 days at 25°C. **(A)** Data are plotted as molar salt or glucose on a logarithmic scale, enabling Hill slope fitting for determination of heteroresistance **(B)** according to the kill-curve gradients. Different shaded symbols represent independent biological replicates. ^∗^*p* < 0.05 according to Welch’s *t-*test. **(C)** Data plotted against the corresponding water activities. Points are means from the three replicates ± SD.

We next tested whether the additional ion-toxicity component of salt versus sugar action, indicated above, may be reflected by quantitative differences in the heterogeneity of resistance (“heteroresistance”) to these solutes. Heteroresistance can be assayed according to the gradients of kill curves (Hewitt et al., 2016), the gradients reflecting the diversity of concentrations at which growth of individual cells becomes inhibited. Kill curves were derived from counts of colony formation at different salt (NaCl) and glucose concentrations in agar (Figure 5A). These particular experiments are performed on agar due to the greater numbers of single colonies that can be readily enumerated (for statistically robust quantitative analysis of heterogeneity) than with static culture in broth. The kill curve data established that extreme resistance to both salt and glucose resides in a small number of resistant cells. The bulk population (100% of cells) was resistant to 0.75 M salt or 1.5 M glucose, but higher concentrations restricted growth to progressively smaller fractions of the population. Only ∼1% of the cell population grew in 1.8 M salt or 2.7 M glucose. These rare resistant colonies grew remarkably slowly, requiring incubation for up to 28 days to detect the colony growth. This is an important consideration for future heterogeneity studies of osmotolerance, as cells with the most extreme resistances may routinely be escaping detection. At more intermediate salt or sugar concentrations, ≥90% of colonies typically appeared within 20 days, whereas <10% appeared between 20 and 28 days. Similar overall patterns of resistance were evident when the data were plotted against water activity at the different salt or glucose concentrations (Figure 5C). Moreover, the relative gradients of the kill curves indicated that heteroresistance was significantly greater for salt than glucose, reflected by the shallower kill curve for salt (Figures 5A,B). Considering the earlier results, this suggests that ion toxicity may elicit an element of cell-cell variation additional to that which can be attributed to osmotic stress. More broadly, the results are consistent with a notion that the degree of heteroresistance can reflect the spectrum of an agent’s mode(s) of action (see section “Discussion”).

Despite comparatively low heteroresistance to glucose after growth in standard conditions (above), pre-adaptation by prior culture for 3 weeks in broth at either 2 M salt or 2.7 M glucose produced a marked elevation of heteroresistance (shallowed kill curve) to glucose, but not salt (Supplementary Figure S3). Cross-resistance was also evident, as expected, indicated by right-ward shift of kill curves conferred in both cases by adaptation to either salt or glucose. Regarding the heteroresistance difference, results suggested that the adaptive response provokes cell-cell variation which may: (i) impact osmo-resistance specifically (glucose), and (ii) be partially suppressed by an additional ion toxicity component (salt). One way in which such a scenario could arise might be where a key protein(s) affecting resistance to ion toxicity becomes more homogeneously expressed during the adaptive response.

### Contribution of *GPD1* to Salt and Sugar Heteroresistance

**FIGURE 6:**
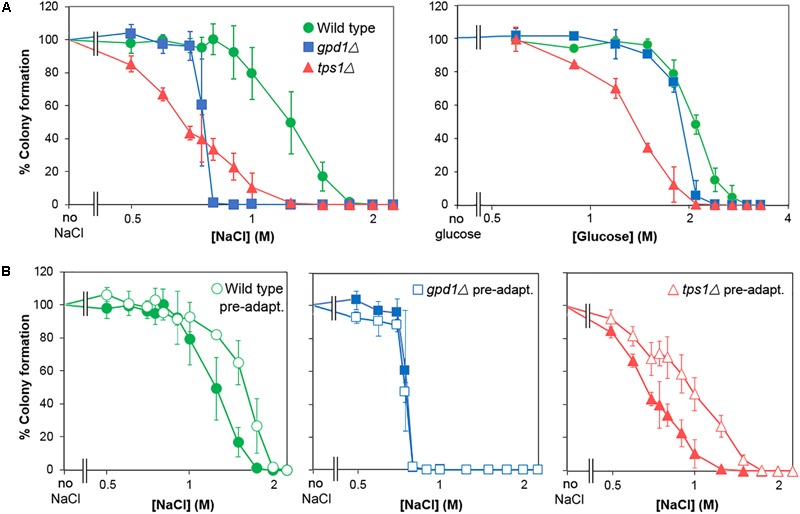
*GPD1* but not *TPS1* is required for heterogeneity of resistance to chronic salt and glucose stresses. **(A)** *S. cerevisiae* BY4741 or isogenic deletion strains *Δgpd1* or *Δtps1* were spread on YEPD agar containing increasing levels of salt (NaCl) (left panel) or glucose (right panel). Colonies were enumerated after 28 days incubation at 25°C. **(B)** Organisms cultured as in **(A)** were pre-treated for 1 h with 0.5 M salt before spreading to YPD agar containing increasing levels of salt. Colonies were enumerated after incubation for 28 days at 25°C. Data are means and SDs from triplicate cultures.

Following the demonstration of adaptation and cross-resistance (above), we used deletion-mutants of *S. cerevisiae* to test the contributions to glucose and salt heteroresistance of key genes known to impact osmo- and halo-tolerance, *GPD1* and *TPS1* (Ansell et al., 1997; Petitjean et al., 2015; Vanacloig-Pedros et al., 2015). We confirmed that the *gpd1Δ* and *tps1Δ* mutants were defective for glycerol and trehalose content, respectively (Supplementary Table S2) and that they showed complete growth inhibition at decreased salt and glucose concentrations (Figure 6A). Strikingly, heteroresistance to salt or glucose was largely eliminated by deletion of *GPD1*: the kill curve for the *gpd1Δ* mutant demonstrated sharp declines in viability over very narrow windows of salt or glucose concentrations. In contrast, *TPS1* deletion had a negligible effect on heteroresistance despite a marked effect on population-averaged resistance, reflected by a left-ward shift of the plot relative to the wild type. Although the IC_50_ (50% inhibitory concentration) for salt was slightly higher in the *gpd1Δ* mutant than the *tps1Δ* mutant, the absence of a resistance tail (elongated curve at low % survival values) at higher salt doses in *gpd1Δ* cells resulted in a concentration giving complete growth inhibition that was greatly decreased compared with the *tps1Δ* mutant; almost the entire *gpd1Δ* population ceased to grow above 0.75 M salt (Figure 6A). Comparison of the adaptive response to salt in the three strains showed that both the wild type and *tps1Δ* mutant had increased salt resistance following pre-adaptation by prior culture with salt (Figure 6B). However, the response was abolished by deletion of *GPD1*. As discussed above, the adaptive response does not alter heteroresistance to salt (Supplementary Figure S3). Therefore, although *GPD1* is evidently required for the adaptive response to salt, the dependency of salt heteroresistance on *GPD1* (Figure 6A) must be rooted before any adaptive response.

**FIGURE 7:**
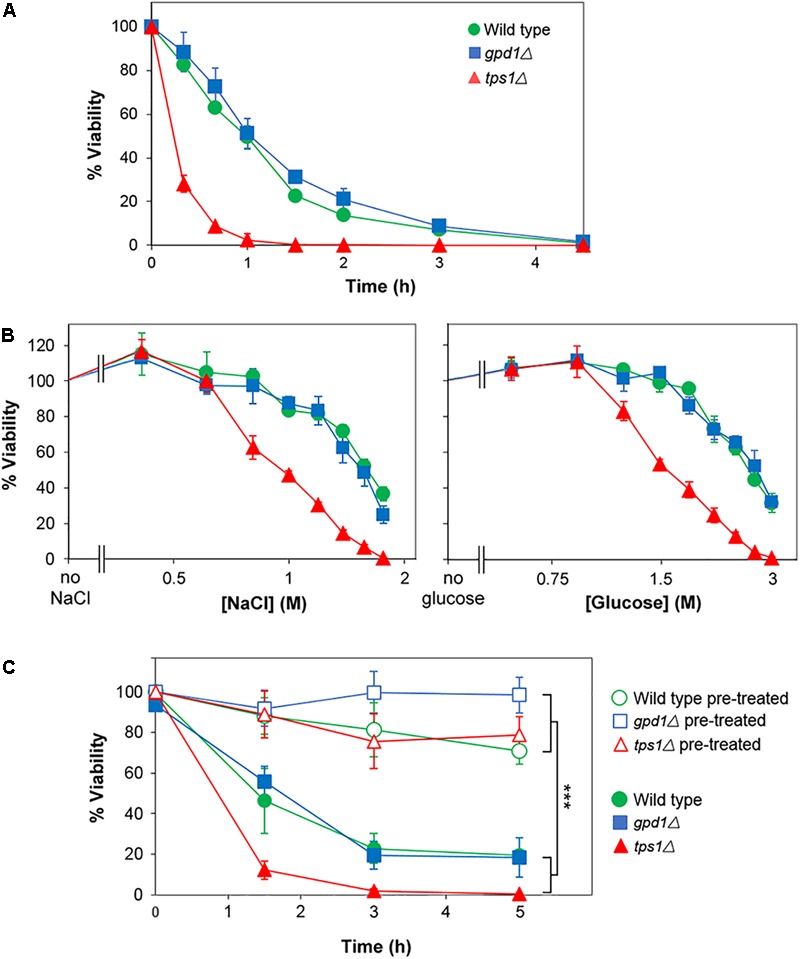
*TPS1* but not *GPD1* confers resistance to acute glucose and salt stresses, without altering heterogeneity. **(A)** *S. cerevisiae* BY4741 (wild type) or isogenic deletion mutants *gpd1Δ* or *tps1Δ* were inoculated to YEPD broth containing 2 M salt (NaCl). Samples were taken at intervals and spread plated to YEPD agar for assessment of viability (colony forming ability). **(B)** Strains were inoculated to YEPD broth supplemented with the indicated salt or glucose concentrations. After 3 h, samples were spread plated to YEPD agar for assessment of viability. **(C)** Cultures were either cultured as normal (open symbols) or with 1 h pre-treatment at 0.5 M salt (NaCl; closed symbols) before inoculation to YEPD broth containing 2 M NaCl. At intervals, samples were spread plated to YEPD agar for assessment of viability. All data are means ± SD from triplicate cultures. ^∗∗∗^*p* < 0.001, unpaired *t-*test.

The above assays reported on chronic stress effects, as they relied on colony formation over an extended period in the presence of salt or glucose. In contrast, acute effects could be captured by assays in which viability is assessed following short term exposure to high salt or glucose. Thus, incubation in broth at 2 M salt resulted in a ∼90% loss of viability within 4 h (according to subsequent assay of colony-forming-ability on agar) (Figure 7A). We compared resistances of the wild type, *gpd1Δ* and *tps1Δ* strains to acute salt or glucose stress. In contrast to the effects seen with chronic stress, *TPS1* but not *GPD1* was required for normal resistance to acute glucose and salt stresses, and in neither mutant was heterogeneity strongly affected (Figure 7B). Furthermore, kinetic studies showed that whereas pre-culture with 0.5 M salt was effective in giving increased resistance to acute salt stress, neither *GPD1* nor *TPS1* were required for that response (Figure 7C). It is notable that the marked effects on heteroresistance were, therefore, confined to chronic stress, as this condition relates more closely to growth on low A_w_ foods and other substrates. The nature of the chronic versus acute stress assays does mean that exposures are on solid versus broth media, which might itself have some impact on resistance-physiology of cells. Nonetheless, the main effects of interest were seen on the solid medium, reinforcing the food relevance. Collectively, the results suggest that Gpd1-dependent glycerol synthesis may be a key and relevant determinant enabling growth of rare yeast subpopulations in low A_w_ conditions, arising from high glucose or salt.

## Discussion

The data presented in this paper establish relationships between salt and sugar resistance across an extensive range of yeast species and strains. Here, we make this dataset available as a reference resource for the wider community. The results showed that, at equivalent water activity to glucose, an additional component to salt toxicity is apparent primarily in osmoresistant yeast strains. In contrast, osmosensitive strains have similar salt and glucose sensitivities. Heteroresistance to salt and sugar was also ubiquitous among the yeast strains, and experiments with *S. cerevisiae* ascribed a major component of such heterogeneity to the glycerol 3-phosphate dehydrogenase encoded by *GPD1*. Rare resistant colonies grew extremely slowly (up to 28 days before detection), emphasizing how extreme resistance may be routinely escaping detection in conventional assays.

The traditional role of high levels of salt and sugar in foods (for example) is to act as dehydration mechanisms for food preservation, resulting in the slowing or inhibition of microbial growth, including food-poisoning bacterial pathogens. Most pathogenic bacteria are sensitive to osmotic stress, few being able to proliferate in foods with a water activity below A_w_ 0.92 (Russell and Gould, 2003). Low water activity appears to be the primary preservation mechanism and some reports have indicated that this is largely irrespective of the chemical nature of the solute (Russell et al., 2003). That inference is supported by the present data only for osmosensitive yeasts, which appeared to be inhibited primarily by the low water activity regardless of whether caused by salts, sugars or other solutes. However, the results for osmoresistant yeasts supported previous reports that salt tends to be more toxic than sugar (Van Eck et al., 1993; Pitt and Hocking, 2009; Dakal et al., 2014). Note, that outcome may be less likely in organisms isolated from high sugar environments but even in soil, for example, sugar has been reported to be more inhibitory in certain bacterial isolates (Fredsgaard et al., 2017). Whereas sugars (and polyols) alter osmotic pressure, salts have the potential to cause changes both in osmotic pressure and ion homeostasis or stress (Dakal et al., 2014). The present comprehensive analysis of diverse yeast species suggests that separate consideration of osmosensitive and osmoresistant organisms could help resolve apparently contradictory reports in the literature.

Many yeast species are moderately osmotolerant and a few yeast species, such as *Z. rouxii*, cause spoilage of sugar conserves, jams, honey and confectionary. The enhanced sensitivity to salt (Na^+^) versus glucose of this organism agrees with reported sensitivity of *Z. rouxii* to salt (Bubnova et al., 2014). A less-extensive, previous survey of 31 yeast species indicated that most of those yeasts were more tolerant of glucose than salt (Van Eck et al., 1993). This is in accord with previous observations that yeasts cause less spoilage in salt-preserved foods (Tokuoka, 1993). Resistance to salt toxicity is largely due to Na^+^ efflux, and it is possible that efflux becomes increasingly ineffective in the high concentrations of salt that inhibit osmotolerant species here (Arino et al., 2010). Other ions such as lithium and cesium exerted a more pronounced level of growth inhibition above that required for osmotic stress. These effects appeared to be fungistatic rather than fungicidal (Figure 2C). Lithium is reported to inhibit specific enzymes (Glaser et al., 1993; Dichtl et al., 1997), while cesium toxicity may be due to action as a potassium analog but it also has been shown to activate the HOG pathway (Avery, 1995; Casagrande et al., 2009). Potassium toxicity is known to be far lower than sodium toxicity as potassium is accumulated in the cytoplasm while sodium is effluxed (Petrezselyova et al., 2010). The present results suggest more similarity in sodium and potassium toxicity (MIC), although that is with regard to external concentrations before taking account of divergent intracellular retention of these ions. Nonetheless, a similar toxicity level would be in keeping with actions based primarily on osmotic stress, whereas additional factors like population heterogeneity have the potential to skew relative measurements of metal-ion toxicity (Bishop et al., 2007). Since high concentrations of salts and sugars are applied in the food industry to prevent spoilage, i.e., to stop the growth of osmotolerant species, the data are consistent with certain salts being more effective than sugars in food preservation.

Resistance to salt and glucose was strongly influenced by population heterogeneity, reflecting wide differences in resistance between individual cells. Heterogeneity was more striking for salt resistance. That may be expected if, as we suggest here, the degree of cell-cell variation in a phenotype is positively correlated with the range of signals contributing to that phenotype; in this case, salt having an additional ion-related toxicity in addition to the osmotic action shared with sugar. Previous studies have established that genetically uniform populations of yeast cells can be very diverse in their resistance to other stressors, with only small sub-populations being highly resistant. This has been demonstrated in resistance to heat (Levy et al., 2012), sorbic acid (Steels et al., 2000; Stratford et al., 2013), drugs (Altamirano et al., 2018; Tindall et al., 2018), and toxic metals (Bishop et al., 2007; Holland et al., 2014), for example. Occasional, rare-cell hyper-resistance means that cell populations which can appear relatively sensitive at intermediate stress levels may persist to very high doses, overtaking the survival of less heterogeneous cell populations (Bishop et al., 2007). Such a phenomenon was evident here in strain comparisons at increasing salt concentrations (Figure 6A). Population heterogeneity to osmotolerance was first reported in the 1980s (MacKenzie et al., 1986) but this field has gained prominence only relatively recently. Heteroresistance has been shown to increase greatly at the onset of stationary phase (Stratford et al., 2014) resulting in enhanced stress survival. Similar increases have been reported for stationary-phase osmotolerance (MacKenzie et al., 1986; Gadd et al., 1987), possibly due to sub-populations of cells containing high trehalose levels.

Previous studies of heteroresistance have not systematically compared the distinct conditions of chronic versus acute stress. The present study revealed that the impact of key treatments on heterogeneity was strikingly dependent on these conditions. The most profound treatment-effects that we observed were on chronic osmotolerance, assessed through long-term growth in the presence of elevated salt or sugar concentrations and a closer reflection of food-relevant growth during storage. Thus, the *GPD1* gene product was required for increased resistance to chronic but not acute salt stress following short term adaptation to lower salt concentrations. This is consistent with distinct strategies needed for survival of short-term killing by acute osmotic stress versus longer-term growth-inhibitory action. Moreover, heteroresistance to chronic (but not acute) osmotic stress was largely eliminated by deletion only of *GPD1*, whereas *TPS1* deletion did not affect heterogeneity. Glycerol synthesis is essential for growth in high levels of salt or sugar. Re-interpreting the work of Edgley and Brown (1983), the present data suggest that there could be considerable diversity between cells in their synthesis of glycerol and that only sub-populations of cells in the wild-type strain are able to synthesize sufficient glycerol to gain the turgor pressure necessary to push out new cell buds, and grow (i.e., not whole population adaptation). These results accord with the previous suggestion that glycerol levels measured in total cell populations may be largely due to a few cells producing excessive amounts (Hohmann, 2008). We propose that single cell glycerol content could be a strong predictor of cell growth or not on low A_w_ foods.

What could drive heterogeneity in Gpd1-dependent glycerol content? Where they’ve been investigated, phenotypes like this that are (dynamically) variable across a genetically uniform cell population [i.e., not assignable to genotypic or epigenetic variation (Avery, 2006; Solieri et al., 2013)] have commonly been traced to noisy gene expression. Gene expression noise can have a stochastic basis (intrinsic) or may be linked to states that are global to single cells but which vary between cells (extrinsic), such as cell cycle stage, cell age, or other deterministic variable that may, for example, differentially trigger signaling cascades (Avery, 2006; Kreft et al., 2017). Such noise can give rise to variation in the abundance of hexose transporter proteins and hexose-responsive transcriptional repression, for example (Welkenhuysen et al., 2017). There is heterogeneity in the transcriptional response of yeast to osmotic stress (Zechner et al., 2012). Furthermore, *GPD1* has been shown to be heterogeneously expressed across a yeast colony, although there is additional micro-environmental variation between cells in that scenario (Traven et al., 2012). As *GPD1* is Hog1 regulated, any noise in the Hog1 pathway could be transmitted to heterogeneous glycerol synthesis. A previous systems-level analysis indicated that the Hog1 response is perfectly adapted to changes in external osmolarity, so predicted to operate with very low noise (Muzzey et al., 2009). However, more recent modeling studies with yeast did report cell to-cell heterogeneity in the Hog1-dependent *GPD1* response, driven at least partly by differences in cell cycle stage and cell size (Spiesser et al., 2016). In addition, cell sub-populations strongly expressing *GPD1* have been described even at low levels of osmotic stress (Aymoz et al., 2016). Their results indicated that *GPD1* expression-noise was not stochastic but that the noise element was only partly dependent on Hog1, implying a Hog1-independent component to the expression variation. The latter interpretation fits with our conclusion that Gpd1-dependent heteroresistance to salt was rooted before any adaptive response (Figures 6 and Supplementary Figure S3).

In contrast to *gpd1Δ* cells, the *tps1Δ* mutant was not affected for heteroresistance, but was defective for (population-averaged) resistance to chronic osmotic stress and, as previously shown (Hounsa et al., 1998), resilience to acute osmotic shock. Trehalose has been reported to protect proteins and membranes from osmotic dehydration (Gadd et al., 1987; Welsh, 2000). The mutant’s sensitivity to desiccation stress has also been ascribed to a novel regulatory function of Tps1 in energy homeostasis rather than trehalose specifically (Petitjean et al., 2015). Nonetheless, heterogeneous response of yeast to heat stress was found to result from the survival of a slow-growing sub-population with high trehalose (Levy et al., 2012).

Adaptation by pre-exposure to low concentrations of salt or glucose may have significant effects on food spoilage. Pre-treatment in 0.5 M salt (30 min–3 h) resulted in close to 100% survival subsequently at high salt or glucose. In foods containing high salt or sugar, the osmotic stress would normally inhibit almost all of the contaminants. However, in a factory producing such foods where diluted salt or sugar was close to the production lines, incidental spoilage yeasts or molds could be subject to osmotic pre-treatment, promoting subsequent survival, growth and spoilage if transferred to the food production. Analysis of the time-line of osmotic stress response has shown that the HOG pathway was initiated between 1 and 10 min and the *GPD1* mRNA peaked after 20 min (Hohmann et al., 2007). As discussed above, such *GPD1*-dependent responses appear to be a key component of adaptive resistance to the chronic osmotic stress that otherwise limits longer-term growth in high salt or high sugar substrates. More broadly, this study establishes new insights to the bases for heteroresistance, as they may differentially impact chronic versus acute stress conditions. Population heterogeneity has received considerable recent attention in the context of adaptation to environment, host or drugs; less so in the context of spoilage processes. Furthermore, microbial applications in other biotechnological bioprocesses are likely also subject to the impacts of population heterogeneity, both in product yields from cells and strain robustness (Hewitt et al., 2016; Binder et al., 2017). Osmotic and other stresses are relevant to diverse industrial processes (Saini et al., 2018), reinforcing the potential implications of heteroresistance for yeast effectiveness in production processes as well as deterioration of foods and other materials.

## Data Availability Statement

Most of the relevant data are included within the manuscript and Supplementary Files. Any additional raw data supporting the conclusions will be made available by the authors on request, without undue reservation, to any qualified researcher.

## Author Contributions

MS, DA, and SA devised the study and drafted the manuscript. MS, MN, and HS performed the experiments.

## Conflict of Interest Statement

The authors declare that the research was conducted in the absence of any commercial or financial relationships that could be construed as a potential conflict of interest.

## Footnotes

1 www.megazyme.com

2 www.aqualab.com

3 www.labcell.com

## References

[B1] Ahmed V. Verma M. K. Gupta S. Mandhan V. Chauhan N. S. (2018). Metagenomic profiling of soil microbes to mine salt stress tolerance genes. *Front. Microbiol.* 9:11. 10.3389/fmicb.2018.00159 29472909 PMC5809485

[B2] Altamirano S. Simmons C. Kozubowski L. (2018). Colony and single cell level analysis of the heterogeneous response of *Cryptococcus neoformans* to fluconazole. *Front. Cell. Infect. Microbiol.* 8:14. 10.3389/fcimb.2018.00203 29971221 PMC6018158

[B3] Ansell R. Granath K. Hohmann S. Thevelein J. M. Adler L. (1997). The two isoenzymes for yeast NAD+-dependent glycerol 3-phosphate dehydrogenase encoded by GPD1 and GPD2 have distinct roles in osmoadaptation and redox regulation. *EMBO J.* 16 2179–2187. 10.1093/emboj/16.9.2179 9171333 PMC1169820

[B4] Arino J. Ramos J. Sychrova H. (2010). Alkali metal cation transport and homeostasis in yeasts. *Microbiol. Mol. Biol. Rev.* 74 95–120. 10.1128/mmbr.00042-09 20197501 PMC2832347

[B5] Avery S. V. (1995). Cesium accumulation by microorganisms – uptake mechanisms, cation competition, compartmentalization, and toxicity. *J. Indust. Microbiol.* 14 76–84. 10.1007/bf01569888 7766213

[B6] Avery S. V. (2006). Microbial cell individuality and the underlying sources of heterogeneity. *Nat. Rev. Microbiol.* 4 577–587. 10.1038/nrmicro1460 16845428

[B7] Aymoz D. Wosika V. Durandau E. Pelet S. (2016). Real-time quantification of protein expression at the single-cell level via dynamic protein synthesis translocation reporters. *Nat. Commun.* 7:12. 10.1038/ncomms11304 27098003 PMC4844680

[B8] Babazadeh R. Lahtvee P. J. Adiels C. B. Goksor M. Nielsen J. B. Hohmann S. (2017). The yeast osmostress response is carbon source dependent. *Sci. Rep.* 7:11. 10.1038/s41598-017-01141-4 28428553 PMC5430539

[B9] Binder D. Drepper T. Jaeger K. E. Delvigne F. Wiechert W. Kohlheyer D. (2017). Homogenizing bacterial cell factories: analysis and engineering of phenotypic heterogeneity. *Metab. Eng.* 42 145–156. 10.1016/j.ymben.2017.06.009 28645641

[B10] Bishop A. L. Rab F. A. Sumner E. R. Avery S. V. (2007). Phenotypic heterogeneity can enhance rare-cell survival in ‘stress-sensitive’ yeast populations. *Mol. Microbiol.* 63 507–520. 10.1111/j.1365-2958.2006.05504.x 17176259

[B11] Bubnova M. Zemancikova J. Sychrova H. (2014). Osmotolerant yeast species differ in basic physiological parameters and in tolerance of non-osmotic stresses. *Yeast* 31 309–321. 10.1002/yea.3024 24962688

[B12] Butinar L. Santos S. Spencer-Martins I. Oren A. Gunde-Cimerman N. (2005). Yeast diversity in hypersaline habitats. *FEMS Microbiol. Lett.* 244 229–234. 10.1016/j.femsle.2005.01.043 15766773

[B13] Casagrande V. Del Vescovo V. Militti C. Mangiapelo E. Frontali L. Negri R. (2009). Cesium chloride sensing and signaling in *Saccharomyces cerevisiae*: an interplay among the HOG and CWI MAPK pathways and the transcription factor Yaf9. *FEMS Yeast Res.* 9 400–410. 10.1111/j.1567-1364.2009.00486.x 19220477

[B14] Dakal T. C. Solieri L. Giudici P. (2014). Adaptive response and tolerance to sugar and salt stress in the food yeast *Zygosaccharomyces rouxii.* *Int. J. Food Microbiol.* 185 140–157. 10.1016/j.ijfoodmicro.2014.05.015 24973621

[B15] Dichtl B. Stevens A. Tollervey D. (1997). Lithium toxicity in yeast is due to the inhibition of RNA processing enzymes. *EMBO J.* 16 7184–7195. 10.1093/emboj/16.23.7184 9384595 PMC1170319

[B16] Dupont S. Beney L. Ferreira T. Gervais P. (2011). Nature of sterols affects plasma membrane behavior and yeast survival during dehydration. *Biochim. Biophys. Acta Biomem.* 1808 1520–1528. 10.1016/j.bbamem.2010.11.012 21081111

[B17] Edgley M. Brown A. D. (1983). Yeast water relations – Physiological changes induced by solute stress in *Saccharomyces cerevisiae* and *Saccharomyces rouxii*. *J. Gen. Microbiol.* 129 3453–3463. 10.1099/00221287-129-11-3453

[B18] Ene I. V. Walker L. A. Schiavone M. Lee K. K. Martin-Yken H. Dague E. (2015). Cell wall remodeling enzymes modulate fungal cell wall elasticity and osmotic stress resistance. *mBio* 6:15. 10.1128/mBio.00986-15 26220968 PMC4551979

[B19] Ferreira C. van Voorst F. Martins A. Neves L. Oliveira R. Kielland-Brandt M. C. (2005). A member of the sugar transporter family, Stl1p is the glycerol/H+ symporter in *Saccharomyces cerevisiae*. *Mol. Biol. Cell* 16 2068–2076. 10.1091/mbc.E04-10-0884 15703210 PMC1073684

[B20] Fredsgaard C. Moore D. B. Al Soudi A. F. Crisler J. D. Chen F. Clark B. C. (2017). Relationships between sucretolerance and salinotolerance in bacteria from hypersaline environments and their implications for the exploration of Mars and the icy worlds. *Int. J. Astrobiol.* 16 156–162. 10.1017/s1473550416000240

[B21] Gadd G. M. Chalmers K. Reed R. H. (1987). The role of trehalose in dehydration resistance of *Saccharomyces cerevisiae*. *FEMS Microbiol. Lett.* 48 249–254. 10.1016/0378-1097(87)90171-6 8461315

[B22] Gena P. Pellegrini-Calace M. Biasco A. Svelto M. Calamita G. (2011). Aquaporin membrane channels: biophysics, classification, functions, and possible biotechnological applications. *Food Biophys.* 6 241–249. 10.1007/s11483-010-9193-9

[B23] Glaser H. U. Thomas D. Gaxiola R. Montrichard F. Surdinkerjan Y. Serrano R. (1993). Salt tolerance and methionine biosynthesis in *Saccharomyces cerevisiae* involve a putative phosphatase gene. *EMBO J.* 12 3105–3110. 10.1002/j.1460-2075.1993.tb05979.x 8393782 PMC413575

[B24] Granot D. Levine A. Dor-Hefetz E. (2003). Sugar-induced apoptosis in yeast cells. *FEMS Yeast Res.* 4 7–13. 10.1016/s1567-1356(03)00154-5 14554192

[B25] Grant W. D. (2004). Life at low water activity. *Philos. Trans. R. Soc. Lond. Ser. B Biol. Sci.* 359 1249–1266. 10.1098/rstb.2004.1502 15306380 PMC1693405

[B26] Hewitt S. K. Foster D. S. Dyer P. S. Avery S. V. (2016). Phenotypic heterogeneity in fungi: importance and methodology. *Fungal Biol. Rev.* 30 176–184. 10.1016/j.fbr.2016.09.002

[B27] Hohmann S. (2002). Osmotic stress signaling and osmoadaptation in yeasts. *Microbiol. Mol. Biol. Rev.* 66 300–372. 10.1128/mmbr.66.2.300-372.2002 12040128 PMC120784

[B28] Hohmann S. (2008). “Integrative analysis of yeast osmoregulation,” in *Stress in Yeasts and Filamentous Fungi* eds Avery S. V. Stratford M. Van West P. (Oxford: Academic) 109–128. 10.1016/S0275-0287(08)80050-1

[B29] Hohmann S. Krantz M. Nordlander B. (2007). “Yeast osmoregulation,” in *Osmosensing and Osmosignaling* eds Haussinger D. Sies H. (San Diego: Elsevier Academic Press Inc),).

[B30] Holland S. L. Reader T. Dyer P. S. Avery S. V. (2014). Phenotypic heterogeneity is a selected trait in natural yeast populations subject to environmental stress. *Environ. Microbiol.* 16 1729–1740. 10.1111/1462-2920.12243 24000788 PMC4231229

[B31] Hosono K. (1992). Effect of salt stress on lipid composition and membrane fluidity of the salt tolerant yeast *Zygosaccharomyces rouxii.* *J. Gen. Microbiol.* 138 91–96. 10.1099/00221287-138-1-91

[B32] Hounsa C. G. Brandt E. V. Thevelein J. Hohmann S. Prior B. A. (1998). Role of trehalose in survival of *Saccharomyces cerevisiae* under osmotic stress. *Microbiology* 144 671–680. 10.1099/00221287-144-3-671 9534237

[B33] Huh G. H. Damsz B. Matsumoto T. K. Reddy M. P. Rus A. M. Ibeas J. I. (2002). Salt causes ion disequilibrium-induced programmed cell death in yeast and plants. *Plant J.* 29 649–659. 10.1046/j.0960-7412.2001.01247.x 11874577

[B34] Kasaai M. R. (2014). Use of water properties in food technology: a global view. *Int. J. Food Prop.* 17 1034–1054. 10.1080/10942912.2011.650339 25439861

[B35] Kreft J. U. Plugge C. M. Prats C. Leveau J. H. J. Zhang W. W. Hellweger F. L. (2017). From genes to ecosystems in microbiology: modeling approaches and the importance of individuality. *Front. Microbiol.* 8:23. 10.3389/fmicb.2017.02299 29230200 PMC5711835

[B36] Kurtzman C. P. (2003). Phylogenetic circumscription of *Saccharomyces, Kluyveromyces* and other members of the Saccharomycetaceae, and the proposal of the new genera *Lachancea, Nakaseomyces, Naumovia, Vanderwaltozyma* and *Zygotorulaspora.* *FEMS Yeast Res.* 4 233–245. 10.1016/s1567-1356(03)00175-2 14654427

[B37] Levy S. F. Ziv N. Siegal M. L. (2012). Bet hedging in yeast by heterogeneous, age-correlated expression of a stress protectant. *PLoS Biol.* 10:16. 10.1371/journal.pbio.1001325 22589700 PMC3348152

[B38] Lievens B. Hallsworth J. E. Pozo M. I. Ben Belgacem Z. Stevenson A. Willems K. A. (2015). Microbiology of sugar-rich environments: diversity, ecology and system constraints. *Environ. Microbiol.* 17 278–298. 10.1111/1462-2920.12570 25041632

[B39] MacKenzie K. F. Blomberg A. Brown A. D. (1986). Water-stress plating hypersensitivity of yeasts. *J. Gen. Microbiol.* 132 2053–2056. 10.1099/00221287-132-7-2053 3540193

[B40] Morris G. J. Winters L. Coulson G. E. Clarke K. J. (1986). Effect of osmotic stress on the ultrastructure and viabilty of the yeast *Saccharomyces cerevisiae*. *J. Gen. Microbiol.* 132 2023–2034. 3540191 10.1099/00221287-132-7-2023

[B41] Muzzey D. Gomez-Uribe C. A. Mettetal J. T. van Oudenaarden A. (2009). A systems-level analysis of perfect adaptation in yeast osmoregulation. *Cell* 138 160–171. 10.1016/j.cell.2009.04.047 19596242 PMC3109981

[B42] Pascual-Ahuir A. Manzanares-Estreder S. Timon-Gomez A. Proft M. (2018). Ask yeast how to burn your fats: lessons learned from the metabolic adaptation to salt stress. *Curr. Genet.* 64 63–69. 10.1007/s00294-017-0724-5 28631015

[B43] Petelenz-Kurdziel E. Kuehn C. Nordlander B. Klein D. Hong K. K. Jacobson T. (2013). Quantitative analysis of glycerol accumulation, glycolysis and growth under hyper osmotic stress. *PLoS Comput. Biol.* 9:10. 10.1371/journal.pcbi.1003084 23762021 PMC3677637

[B44] Petitjean M. Teste M. A. Francois J. M. Parrou J. L. (2015). Yeast tolerance to various stresses relies on the trehalose-6P Synthase (Tps1) protein, not on trehalose. *J. Biol. Chem.* 290 16177–16190. 10.1074/jbc.M115.653899 25934390 PMC4481218

[B45] Petrezselyova S. Zahradka J. Sychrova H. (2010). *Saccharomyces cerevisiae* BY4741 and W303-1A laboratory strains differ in salt tolerance. *Fungal Biol.* 114 144–150. 10.1016/j.funbio.2009.11.002 20960970

[B46] Pitt J. I. Hocking A. D. (2009). *Fungi and Food Spoilage, Third Edition*. New York, NY: Springer 10.1007/978-0-387-92207-2

[B47] Rodriguez-Vargas S. Sanchez-Garcia A. Martinez-Rivas J. M. Prieto J. A. Randez-Gil F. (2007). Fluidization of membrane lipids enhances the tolerance of *Saccharomyces cerevisiae* to freezing and salt stress. *Appl. Environ. Microbiol.* 73 110–116. 10.1128/aem.01360-06 17071783 PMC1797130

[B48] Russell N. J. Gould G. W. (2003). “Major preservation technologies,” in *Food preservatives* 2nd Edn eds Russell N. J. Gould G. W. (New York, NY: Kluwer Academic) 14–24.

[B49] Russell N. J. Leistner L. Gould G. W. (2003). “Solutes and low water activity,” in *Food preservatives* 2nd Edn eds Russell N. J. Gould G. W. (New York, NY: Kluwer Academic) 119–145.

[B50] Sabir F. Loureiro-Dias M. C. Soveral G. Prista C. (2017). Functional relevance of water and glycerol channels in *Saccharomyces cerevisiae*. *FEMS Microbiol. Lett.* 364:fnx080. 10.1093/femsle/fnx080 28430948

[B51] Saini P. Beniwal A. Kokkiligadda A. Vij S. (2018). Response and tolerance of yeast to changing environmental stress during ethanol fermentation. *Proc. Biochem.* 72 1–12. 10.1016/j.procbio.2018.07.001

[B52] Silva R. D. Sotoca R. Johansson B. Ludovico P. Sansonetty F. Silva M. T. (2005). Hyperosmotic stress induces metacaspase- and mitochondria-dependent apoptosis in *Saccharomyces cerevisiae*. *Mol. Microbiol.* 58 824–834. 10.1111/j.1365-2958.2005.04868.x 16238630

[B53] Smith D. A. Morgan B. A. Quinn J. (2010). Stress signalling to fungal stress-activated protein kinase pathways. *FEMS Microbiol. Lett.* 306 1–8. 10.1111/j.1574-6968.2010.01937.x 20345377 PMC3644883

[B54] Solieri L. Dakal T. C. Bicciato S. (2014). Quantitative phenotypic analysis of multistress response in *Zygosaccharomyces rouxii* complex. *FEMS Yeast Res.* 14 586–600. 10.1111/1567-1364.12146 24533625

[B55] Solieri L. Dakal T. C. Croce M. A. Giudici P. (2013). Unravelling genomic diversity of *Zygosaccharomyces rouxii* complex with a link to its life cycle. *FEMS Yeast Res.* 13 245–258. 10.1111/1567-1364.12027 23279556

[B56] Spiesser T. Kuhn C. Krantz M. Klipp E. (2016). The MYpop toolbox: putting yeast stress responses in cellular context on single cell and population scales. *Biotechnol. J.* 11 1158–1168. 10.1002/biot.201500344 26952199

[B57] Steels H. James S. A. Roberts I. N. Stratford M. (2000). Sorbic acid resistance: the inoculum effect. *Yeast* 16 1173–1183. 10.1002/1097-0061(20000930)16:13<1173::AID-YEA617>3.0.CO;2-8 10992281

[B58] Stratford M. Steels H. Nebe-von-Caron G. Avery S. V. Novodvorska M. Archer D. B. (2014). Population heterogeneity and dynamics in starter culture and lag phase adaptation of the spoilage yeast *Zygosaccharomyces bailii* to weak acid preservatives. *Int. J. Food Microbiol.* 181 40–47. 10.1016/j.ijfoodmicro.2014.04.017 24813627 PMC4058750

[B59] Stratford M. Steels H. Nebe-von-Caron G. Novodvorska M. Hayer K. Archer D. B. (2013). Extreme resistance to weak-acid preservatives in the spoilage yeast *Zygosaccharomyces bailii*. *Int. J. Food Microbiol.* 166 126–134. 10.1016/j.ijfoodmicro.2013.06.025 23856006 PMC3759830

[B60] Talemi S. R. Tiger C. F. Andersson M. Babazadeh R. Welkenhuysen N. Klipp E. (2016). Systems level analysis of the yeast osmo-stat. *Sci. Rep.* 6:12. 10.1038/srep30950 27515486 PMC4981887

[B61] Tamas M. J. Luyten K. Sutherland F. C. W. Hernandez A. Albertyn J. Valadi H. (1999). Fps1p controls the accumulation and release of the compatible solute glycerol in yeast osmoregulation. *Mol. Microbiol.* 31 1087–1104. 10.1046/j.1365-2958.1999.01248.x 10096077

[B62] Tindall S. M. Vallieres C. Lakhani D. H. Islahudin F. Ting K. N. Avery S. V. (2018). Heterologous expression of a novel drug transporter from the malaria parasite alters resistance to quinoline antimalarials. *Sci. Rep.* 8:11. 10.1038/s41598-018-20816-0 29410428 PMC5802821

[B63] Tokuoka K. (1993). Sugar-tolerant and salt-tolerant yeasts. *J. Appl. Bacteriol.* 74 101–110. 10.1111/j.1365-2672.1993.tb03002.x

[B64] Traven A. Janicke A. Harrison P. Swaminathan A. Seemann T. Beilharz T. H. (2012). Transcriptional profiling of a yeast colony provides new insight into the heterogeneity of multicellular fungal communities. *PLoS One* 7:11. 10.1371/journal.pone.0046243 23029448 PMC3460911

[B65] Vallieres C. Raulo R. Dickinson M. Avery S. V. (2018). Novel combinations of agents targeting translation that synergistically inhibit fungal pathogens. *Front. Microbiol.* 9:2355. 10.3389/fmicb.2018.02355 30349511 PMC6186996

[B66] Van Eck J. H. Prior B. A. Brandt E. V. (1993). The water relations of growth and polyhydroxy alcohol production by ascomycetous yeasts. *J. Gen. Microbiol.* 139 1047–1054. 10.1099/00221287-139-5-1047

[B67] Vanacloig-Pedros E. Bets-Plasencia C. Pascual-Ahuir A. Proft M. (2015). Coordinated gene regulation in the initial phase of salt stress adaptation. *J. Biol. Chem.* 290 10163–10175. 10.1074/jbc.M115.637264 25745106 PMC4400332

[B68] Vyrides I. Stuckey D. C. (2017). Compatible solute addition to biological systems treating waste/wastewater to counteract osmotic and other environmental stresses: a review. *Crit. Rev. Biotechnol.* 37 865–879. 10.1080/07388551.2016.1266460 28043169

[B69] Welkenhuysen N. Borgqvist J. Backman M. Bendrioua L. Goksor M. Adiels C. B. (2017). Single-cell study links metabolism with nutrient signaling and reveals sources of variability. *BMC Syst. Biol.* 11:10. 10.1186/s12918-017-0435-z 28583118 PMC5460408

[B70] Welsh D. T. (2000). Ecological significance of compatible solute accumulation by micro-organisms: from single cells to global climate. *FEMS Microbiol. Rev.* 24 263–290. 10.1111/j.1574-6976.2000.tb00542.x 10841973

[B71] Zechner C. Ruess J. Krenn P. Pelet S. Peter M. Lygeros J. (2012). Moment-based inference predicts bimodality in transient gene expression. *Proc. Natl. Acad. Sci. U.S.A.* 109 8340–8345. 10.1073/pnas.1200161109 22566653 PMC3361437

