# Supplementary material for: Extreme Osmotolerance and Halotolerance in Food-Relevant Yeasts and the Role of Glycerol-Dependent Cell Individuality
Source: Front Microbiol. 2019 Jan 9;9:3238. doi: 10.3389/fmicb.2018.03238 (PMC6333755; doi:10.3389/fmicb.2018.03238)
Supplement: Supplementary file 1 [file Data_Sheet_1.PDF]

Table S1 Yeast species and their origins, tested for their resistance to sugar (glucose) and salt (NaCl) in YEPD pH 4.0 after 2 weeks incubation at 25°C. Strains numbers refer to the Mologic yeast collection. In the salt tests YEPD contained 0.11 M glucose in addition to the salt. In the glucose tests the YEPD contained the listed concentration of glucose only. The water activity (Aw) was tested in the YEPD medium.

			**MIC (M)**		**A_w**	
**Yeast Species**	**Strain**	**Origin**	**Glucose**	**NaCl**	**Glucose**	**NaCl**
Barnettozyma californica	376	Turkey	1.8	1.1	0.954	0.957
Barnettozyma californica	538	Brazil	2.25	1	0.938	0.96
Barnettozyma sp.nov.	486	Brazil	2.1	0.9	0.944	0.964
Barnettozyma sp.nov.	497	Brazil	2	1.2	0.948	0.954
Barnettozyma subsufficiens	539	Brazil	1.7	1.55	0.957	0.94
Brettanomyces naardenensis	27	UK	2.4	1.2	0.933	0.954
Brettanomyces naardenensis	129	UK	1.6	1	0.96	0.96
Brettanomyces naardenensis	131	UK	1.5	1	0.963	0.96
Candida aaseri	440	Turkey	2.75	2.85	0.92	0.884
Candida aaseri	537	Brazil	2.8	0.9	0.916	0.964
Candida albicans	212	Netherlands	3.3	3	0.892	0.876
Candida albicans	309	Italy	4.05	2.05	0.844	0.919
Candida apicola	530	Brazil	4.5	1.4	0.807	0.946
Candida boidinii	166	Netherlands	2.5	1.8	0.928	0.93
Candida boidinii	204	Netherlands	2.5	1.2	0.928	0.954
Candida boidinii	295	UK	2.25	1.7	0.938	0.935
Candida boidinii	307	Belgium	3.6	2	0.874	0.921
Candida boidinii	368	Turkey	2.7	1.65	0.921	0.937
Candida boidinii	411	Turkey	2.75	1.55	0.92	0.94
Candida boidinii	420	Turkey	2.6	1.9	0.925	0.926
Candida boidinii	430	Turkey	2.85	2.05	0.914	0.919
Candida boidinii	464	Russia	2.7	1.55	0.921	0.94
Candida boidinii	487	Brazil	2.5	1.7	0.928	0.935
Candida boidinii	527	Brazil	2.8	2.25	0.916	0.911
Candida boidinii	557	Brazil	2.75	2.2	0.92	0.913
Candida boidinii	603	Russia	2.65	2	0.923	0.921
Candida boidinii	637	UK	2.35	1.6	0.935	0.939
Candida carpophila	350	Thailand	3.8	2.55	0.861	0.898
Candida davenportii	220	Netherlands	3.3	1.7	0.892	0.935
Candida diddensiae	355	Thailand	3.9	1.8	0.854	0.93
Candida diddensiae	553	Brazil	4	3.15	0.849	0.87
Candida diddensiae	611	Russia	3.8	2.85	0.861	0.884
Candida etchellsii	240	Netherlands	4.75	4	0.785	0.826
Candida inconspicua sister sp.	457	Russia	2.2	1.65	0.94	0.937
Candida intermedia	382	Turkey	2.75	2.2	0.92	0.913
Candida intermedia	NCYC 2504		3	2.05	0.907	0.919
Candida intermedia	NCYC 2531		3	2.4	0.907	0.904
Candida magnoliae	M1	UK	5	3.65	0.756	0.845
Candida melibiosica	328	Thailand	2.8	2.42	0.916	0.903
Candida melibiosica	345	Thailand	2.9	2.5	0.911	0.9
Candida melibiosica	346	Thailand	2.95	2.55	0.909	0.898
Candida natalensis	518	Brazil	2.85	1.35	0.914	0.948

Yeast Species	Strain	Origin	MIC (M)		A_w	
			Glucose	NaCl	Glucose	NaCl
Candida neerlandica sister sp.	493	Brazil	2.8	1.9	0.916	0.926
Candida norwegica	215	Netherlands	3.4	3.2	0.886	0.867
Candida oleophila	147	Netherlands	3.75	2.7	0.864	0.891
Candida oleophila	206	Netherlands	3.2	2.7	0.897	0.891
Candida oleophila	467	Russia	3.3	2.65	0.892	0.893
Candida oleophila	598	Russia	3.5	2.6	0.88	0.896
Candida orthopsilosis	238	Thailand	3.75	3.4	0.864	0.858
Candida orthopsilosis	358	Thailand	3.8	3.15	0.861	0.87
Candida orthopsilosis	517	Brazil	4.15	3.4	0.836	0.858
Candida parapsilosis	67	UK	3.5	3.2	0.88	0.867
Candida parapsilosis	68	UK	3.5	3.2	0.88	0.867
Candida parapsilosis	69	UK	3.5	3.4	0.88	0.858
Candida parapsilosis	138	France	4	3.4	0.849	0.858
Candida parapsilosis	157	Netherlands	3.6	3.6	0.874	0.847
Candida parapsilosis	163	UK	3.4	3.5	0.886	0.852
Candida parapsilosis	164	UK	3.9	3.4	0.854	0.858
Candida parapsilosis	203	Ghana	3.4	3.9	0.886	0.832
Candida parapsilosis	283	UK	3.5	3.2	0.88	0.867
Candida parapsilosis	384	Turkey	3.65	3.25	0.871	0.865
Candida parapsilosis	408	Turkey	3.05	3.2	0.904	0.867
Candida parapsilosis	432	Turkey	3.9	3.15	0.854	0.87
Candida parapsilosis	451	Russia	3.2	3.3	0.897	0.862
Candida parapsilosis	484	Brazil	4.1	3.05	0.84	0.874
Candida parapsilosis	583	S Africa	3.9	3.15	0.854	0.87
Candida parapsilosis	589	Belgium	4	3	0.849	0.876
Candida parapsilosis	625	Russia	3.15	3.4	0.9	0.858
Candida parapsilosis	630	Germany	4	2.9	0.849	0.882
Candida parapsilosis	644	UK	3.5	3.1	0.88	0.872
Candida parapsilosis	662	UK	3.25	2.95	0.895	0.879
Candida parapsilosis	672	UK	3.35	3.1	0.889	0.872
Candida parapsilosis	679	UK	3.25	3.15	0.895	0.87
Candida parapsilosis	682	UK	2.85	3.05	0.914	0.874
Candida pararugosa	122	UK	3.5	2.2	0.88	0.913
Candida pseudoglaebosa	223	Netherlands	2.5	1.8	0.928	0.93
Candida pseudointermedia	79	France	3	2.4	0.907	0.904
Candida pseudointermedia	153	Netherlands	2.9	1.9	0.911	0.926
Candida pseudointermedia	211	Netherlands	2.6	2.5	0.925	0.9
Candida pseudointermedia	227	Netherlands	2.7	2.3	0.921	0.909
Candida pseudointermedia	235	Netherlands	3.1	2.5	0.902	0.9
Candida pseudointermedia	246	Netherlands	2.9	2.3	0.911	0.909
Candida pseudointermedia	281	France	2.75	2.4	0.92	0.904
Candida pseudointermedia	327	Thailand	2.95	2.15	0.909	0.915
Candida pseudointermedia	332	Thailand	2.9	2.2	0.911	0.913
Candida pseudointermedia	340	Thailand	3	2.05	0.907	0.919
Candida pseudointermedia	351	Thailand	2.9	2.25	0.911	0.911
Candida pseudointermedia	372	Turkey	3.05	2.1	0.904	0.917
Candida pseudointermedia	407	Turkey	3	2.3	0.907	0.909
Candida pseudointermedia	416	Turkey	3	2.35	0.907	0.907
Candida pseudointermedia	419	Turkey	3	2.25	0.907	0.911

Yeast Species	Strain	Origin	MIC (M)		A_w	
			Glucose	NaCl	Glucose	NaCl
Candida pseudointermedia	425	Turkey	2.95	2.35	0.909	0.907
Candida pseudointermedia	460	Russia	3	2.6	0.907	0.896
Candida pseudointermedia	495	Brazil	3	2.15	0.907	0.915
Candida pseudointermedia	496	Brazil	2.9	2.3	0.911	0.909
Candida pseudointermedia	501	Brazil	3.2	2.45	0.897	0.902
Candida pseudointermedia	519	Brazil	3	0.8	0.907	0.968
Candida pseudointermedia	528	Brazil	3	1.05	0.907	0.959
Candida pseudointermedia	552	Brazil	3.15	2.6	0.9	0.896
Candida pseudointermedia	556	Brazil	3	2.05	0.907	0.919
Candida pseudointermedia	590	France	2.7	2.2	0.921	0.913
Candida pseudointermedia	591	France	2.7	2.15	0.921	0.915
Candida pseudointermedia	606	Russia	3	2.5	0.907	0.9
Candida pseudointermedia	629	Netherlands	3	2.3	0.907	0.909
Candida pseudointermedia	639	UK	2.9	2.25	0.911	0.911
Candida pseudointermedia	640	UK	3	2.3	0.907	0.909
Candida pseudointermedia	680	UK	3	2.2	0.907	0.913
Candida pseudointermedia	NCYC 2610		3.3	2.3	0.892	0.909
Candida pseudointermedia	NCYC 3278		3.25	2.25	0.895	0.911
Candida pseudolambica	400	Turkey	2.1	1.45	0.944	0.944
Candida pseudolambica	435	Turkey	2	1.4	0.948	0.946
Candida pseudolambica	449	Russia	1.9	1.45	0.951	0.944
Candida pseudolambica	485	Brazil	2.65	1.7	0.923	0.935
Candida pseudolambica	525	Brazil	1.95	1.65	0.95	0.937
Candida pseudolambica	526	Brazil	1.8	1.65	0.954	0.937
Candida pseudolambica	599	Russia	2.25	1.55	0.938	0.94
Candida pseudolambica	605	Russia	2.2	1.8	0.94	0.93
Candida pseudolambica	618	Russia	2.2	1.45	0.94	0.944
Candida pseudolambica	627	Russia	2.25	1.5	0.938	0.942
Candida rugosa	353	Thailand	3.15	2.55	0.9	0.898
Candida sake	322	UK	2.9	1.6	0.911	0.939
Candida sake	323	UK	2.95	2.1	0.909	0.917
Candida silvae	626	Russia	2.45	1.95	0.931	0.924
Candida sojae	121	UK	3.5	2.4	0.88	0.904
Candida sojae	126	France	3.5	2.4	0.88	0.904
Candida sojae	165	Poland	3.25	2.7	0.895	0.891
Candida sojae	245	Netherlands	2.9	2.4	0.911	0.904
Candida sojae	312	Brazil	3.6	2.3	0.874	0.909
Candida sojae	375	Turkey	2.9	2.42	0.911	0.903
Candida sojae	414	Turkey	3	2.45	0.907	0.902
Candida sojae	434	Turkey	2.8	2.35	0.916	0.907
Candida sojae	439	Turkey	2.8	2.15	0.916	0.915
Candida sojae	446	France	3	2.3	0.907	0.909
Candida sojae	483	Brazil	2.95	2.3	0.909	0.909
Candida sojae	601	Russia	2.9	2.3	0.911	0.909
Candida sojae	608	Russia	2.9	2.5	0.911	0.9
Candida sorbophila	168	Thailand	2.7	2.45	0.921	0.902
Candida sorbophila	NCYC 173		2.8	2.4	0.916	0.904
Candida sorbosivorans	479	Brazil	4.3	4	0.825	0.826
Candida sp.	383	Turkey	2.85	2.45	0.914	0.902

Yeast Species	Strain	Origin	MIC (M)		A_w	
			Glucose	NaCl	Glucose	NaCl
Candida sp.nov.	397	China	5	3.8	0.756	0.836
Candida sp.nov.	452	Russia	2.85	1.9	0.914	0.926
Candida sp.nov.	524	Brazil	4.2	3.2	0.832	0.867
Candida sp.nov.	577	Germany	3.5	2.25	0.88	0.911
Candida sp.nov.	674	UK	2.8	1.8	0.916	0.93
Candida sp.nov.	675	UK	2.85	1.85	0.914	0.928
Candida sp.nov.	681	UK	2.7	1.75	0.921	0.933
Candida sp.nov.	683	UK	2.65	1.85	0.923	0.928
Candida tropicalis	342	Thailand	3	2.3	0.907	0.909
Candida tropicalis	344	Thailand	3.15	2.35	0.9	0.907
Candida tropicalis	352	Thailand	3.2	2.3	0.897	0.909
Candida tropicalis	369	Turkey	3	2.15	0.907	0.915
Candida tropicalis	389	Turkey	3	2.25	0.907	0.911
Candida tropicalis	417	Turkey	3.15	2.4	0.9	0.904
Candida tropicalis	428	Turkey	2.9	2.7	0.911	0.891
Candida tropicalis	520	Brazil	3.15	2.9	0.9	0.882
Candida tropicalis sister sp.	377	Turkey	3.15	2.5	0.9	0.9
Candida tropicalis sister sp.	492	Brazil	3.2	2.35	0.897	0.907
Candida vartiovaarae	622	Russia	1.8	1.45	0.954	0.944
Candida versitalis	396	China	4.5	3.45	0.807	0.855
Candida wyomingensis	617	Russia	2.8	1.95	0.916	0.924
Candida zeylandoides	26	UK	3	2.8	0.907	0.886
Clavispora lusitania	53	Israel	3.15	2.4	0.9	0.904
Clavispora lusitania	74	France	3.5	2.6	0.88	0.896
Clavispora lusitania	110	Argentina	3	2.4	0.907	0.904
Clavispora lusitania	225	Netherlands	3.1	2.7	0.902	0.891
Clavispora lusitania	399	Germany	2.9	2.45	0.911	0.902
Clavispora lusitania	436	Turkey	3.05	2.3	0.904	0.909
Clavispora lusitania	443	Turkey	3.2	2.55	0.897	0.898
Clavispora lusitania	444	Turkey	2.8	2.35	0.916	0.907
Clavispora lusitania	445	Turkey	3.1	2.65	0.902	0.893
Clavispora lusitania	461	Russia	2.8	2.5	0.916	0.9
Clavispora lusitania	462	Russia	2.9	2.5	0.911	0.9
Clavispora lusitania	481	Brazil	3.2	2.25	0.897	0.911
Clavispora lusitania	531	Brazil	3	1.1	0.907	0.957
Clavispora lusitania	559	Brazil	2.9	2.6	0.911	0.896
Clavispora lusitania	643	UK	3.2	2.55	0.897	0.898
Clavispora lusitania	655	UK	3.3	2.3	0.892	0.909
Clavispora lusitania	671	UK	2.7	2.2	0.921	0.913
Clavispora lusitania	53a	Israel	3.7	2	0.868	0.921
Clavispora lusitania	NCYC 3268		3.75	2.3	0.864	0.909
Cryptococcus albidosimilis	361	Turkey	1.5	1.2	0.963	0.954
Cryptococcus albidus	144	Netherlands	2	1.4	0.948	0.946
Cryptococcus albidus	216	Netherlands	2.4	1.2	0.933	0.954
Cryptococcus albidus	660	UK	1.7	2.25	0.957	0.911
Cryptococcus albidus	673	UK	1.8	1.45	0.954	0.944
Cryptococcus albidus	NCYC 445		2.5	2	0.928	0.921
Cryptococcus cylindricus	82	UK	3	1.8	0.907	0.93
Cryptococcus diffluens	600	Russia	2.4	1.95	0.933	0.924

Yeast Species	Strain	Origin	MIC (M)		A_w	
			Glucose	NaCl	Glucose	NaCl
Cryptococcus flavescens	214	Netherlands	2.8	2	0.916	0.921
Cryptococcus humicola	490	Brazil	2.85	1.2	0.914	0.954
Cryptococcus laurentii	60	UK	3	2.2	0.907	0.913
Cryptococcus laurentii	77	France	3	2.4	0.907	0.904
Cryptococcus laurentii	78	France	3	2.4	0.907	0.904
Cryptococcus laurentii	99	UK	3	1.2	0.907	0.954
Cryptococcus laurentii	341	Thailand	2.55	2.2	0.927	0.913
Cryptococcus laurentii	347	Thailand	3.1	2.35	0.902	0.907
Cryptococcus laurentii	360	Turkey	3.1	2.02	0.902	0.92
Cryptococcus laurentii	433	Turkey	2.95	2.15	0.909	0.915
Cryptococcus laurentii	480	Brazil	2.9	2.1	0.911	0.917
Cryptococcus laurentii	546	Brazil	2.6	2.3	0.925	0.909
Cryptococcus laurentii	607	Russia	3	2	0.907	0.921
Cryptococcus laurentii	609	Russia	2.95	2.05	0.909	0.919
Cryptococcus laurentii sister sp	404	Turkey	2.85	1.7	0.914	0.935
Cryptococcus laurentii sister sp	580	S Africa	2.8	2.15	0.916	0.915
Cryptococcus laurentii sister sp	664	UK	1.4	2.25	0.966	0.911
Cryptococcus liquefaciens	349	Thailand	2.4	2.6	0.933	0.896
Cryptococcus liquefaciens	357	Thailand	2.15	2.3	0.942	0.909
Cryptococcus liquefaciens	402	Turkey	2.2	1.8	0.94	0.93
Cryptococcus liquefaciens	418	Turkey	2.15	2.05	0.942	0.919
Cryptococcus liquefaciens	424	Turkey	2.45	2.1	0.931	0.917
Cryptococcus magnus	422	Turkey	2.35	1.7	0.935	0.935
Cryptococcus magnus	465	Russia	2.2	1.8	0.94	0.93
Cryptococcus magnus	628	Russia	2.3	1.65	0.937	0.937
Cryptococcus magnus	645	UK	2.2	2	0.94	0.921
Cryptococcus magnus	666	UK	2.25	1.45	0.938	0.944
Cryptococcus nyarrowii sister sp.	85	UK	3.5	3.6	0.88	0.847
Cryptococcus ramirezgomezianus	145	Netherlands	2.4	1.3	0.933	0.95
Cryptococcus saitou	234	Netherlands	2.5	1.9	0.928	0.926
Cryptococcus saitou	448	Russia	2.25	1.85	0.938	0.928
Cryptococcus saitou	584	S Africa	2	1.7	0.948	0.935
Cryptococcus saitou	663	UK	1.85	1.8	0.952	0.93
Cryptococcus sp.nov.	84	UK	4	2	0.849	0.921
Cryptococcus sp.nov.	547	Brazil	2.6	1.3	0.925	0.95
Cryptococcus sp.nov.	548	Brazil	2.85	2.05	0.914	0.919
Cryptococcus uzbekistanensis	586	S Africa	1.8	1.45	0.954	0.944
Cryptococcus victoriae sister sp.	665	UK	1.4	2.25	0.966	0.911
Debaryomyces hansenii	146	Netherlands	3.9	1.9	0.854	0.926
Debaryomyces hansenii	403	Turkey	4.1	2.85	0.84	0.884
Debaryomyces hansenii	437	Turkey	3.95	3.1	0.851	0.872
Debaryomyces hansenii	450	Russia	3.9	3.4	0.854	0.858
Debaryomyces hansenii	657	UK	3.85	3.4	0.858	0.858
Debaryomyces hansenii	NCYC 9		3	2.4	0.907	0.904
Debaryomyces hansenii var *fabryii*	100	UK	4	3.4	0.849	0.858
Debaryomyces hansenii var *fabryii*	558	Brazil	3.85	3.3	0.858	0.862
Dekkera anomala	247	Belgium	1.5	1.3	0.963	0.95
Dekkera anomala	502	UK	2.1	1.25	0.944	0.952
Dekkera anomala	506	UK	2.05	1.4	0.946	0.946

Yeast Species	Strain	Origin	MIC (M)		A_w	
			Glucose	NaCl	Glucose	NaCl
Dekkera anomala	247	Belgium	1.5	1.3	0.963	0.95
Dekkera anomala	506	UK	2.05	1.4	0.946	0.946
Dekkera bruxellensis	148	Netherlands	1.8	1.4	0.954	0.946
Dekkera bruxellensis	306	Belgium	1.7	1.2	0.957	0.954
Dekkera bruxellensis	311	UK	2	1.1	0.948	0.957
Dekkera bruxellensis	319	Belgium	2.05	1.1	0.946	0.957
Dekkera bruxellensis	325	Belgium	2.1	1.35	0.944	0.948
Dekkera bruxellensis	326	Belgium	2.15	1.3	0.942	0.95
Dekkera bruxellensis	507	UK	2.15	1.55	0.942	0.94
Dekkera bruxellensis	NCYC 823		1.5	1	0.963	0.96
Filobasidiella neoformans	545	Brazil	2.7	2.7	0.921	0.891
Filobasidium uniguttulatum	230	Netherlands	2.7	2.8	0.921	0.886
Hanseniaspora guilliermondii	NRRL 1625		3.35	2.1	0.889	0.917
Hanseniaspora meyeri	81	UK	3.5	2.2	0.88	0.913
Hanseniaspora meyeri	127	France	3.5	2.4	0.88	0.904
Hanseniaspora meyeri	155	Netherlands	3.3	2.7	0.892	0.891
Hanseniaspora occidentalis	NRRL 7946		2.9	1.45	0.911	0.944
Hanseniaspora osmophila	NRRL 1613		3.8	1.5	0.861	0.942
Hanseniaspora uvarum	226	Netherlands	3.35	2.4	0.889	0.904
Hanseniaspora uvarum	321	UK	3.2	2.45	0.897	0.902
Hanseniaspora uvarum	646	UK	3.4	2.4	0.886	0.904
Hanseniaspora uvarum	NRRL 1614		3.4	2	0.886	0.921
Hanseniaspora uvarum	NRRL 1626		3.1	2.05	0.902	0.919
Hanseniaspora vineae	NRRL17529		3.4	1.4	0.886	0.946
Kazachstania barnettii	57	UK	2.5	2	0.928	0.921
Kazachstania exigua	23	UK	3	2	0.907	0.921
Kazachstania exigua	55	UK	3	1.8	0.907	0.93
Kazachstania exigua	152	Netherlands	2.75	2.1	0.92	0.917
Kazachstania kunashirensis	NCYC 2702		2.5	1.6	0.928	0.939
Kazachstania martiniae	NCYC 2703		2.25	1.45	0.938	0.944
Kazachstania servazzii	58	UK	3	2.2	0.907	0.913
Kazachstania servazzii	NCYC 2577		3.05	1.9	0.904	0.926
Kazachstania unispora	NCYC 971		2.45	1.85	0.931	0.928
Kloeckera linderi	NRRL17531		2.4	1.4	0.933	0.946
Kluyveromyces marxianus	562	UK	2.4	1.8	0.933	0.93
Kodamaea ohmeri	348	Thailand	4.35	2.95	0.821	0.879
Kodamaea ohmeri	535	Brazil	4.1	1.35	0.84	0.948
Komagataella pastoris	219	Netherlands	2.4	1.8	0.933	0.93
Kregervanrija fluxuum	504	UK	2.45	1.5	0.931	0.942
Lachancea cidri	NCYC 2875		3.5	2.2	0.88	0.913
Lachancea fermentati	NCYC 2508		3	2	0.907	0.921
Lindnera fabianii	534	Brazil	3.25	1.5	0.895	0.942
Lindnera jadinii	365	Turkey	1.9	1.4	0.951	0.946
Lindnera jadinii	477	Brazil	2.05	1.3	0.946	0.95
Lindnera jadinii	604	Russia	2.1	1.75	0.944	0.933
Lindnera jadinii sister sp.	209	Netherlands	2.5	1.1	0.928	0.957
Lodderomyces elongisporus	373	Turkey	3.35	2.85	0.889	0.884
Metschnikowia sp.nov.	229	Netherlands	2.75	1.5	0.92	0.942
Metschnikowia sp.nov.	536	Brazil	3	1.1	0.907	0.957

Yeast Species	Strain	Origin	MIC (M)		A_w	
			Glucose	NaCl	Glucose	NaCl
Meyerozyma guilliermondii	224	Netherlands	3.2	3.1	0.897	0.872
Meyerozyma guilliermondii	343	Thailand	3.4	2.75	0.886	0.889
Meyerozyma guilliermondii	370	Turkey	3.1	2.5	0.902	0.9
Meyerozyma guilliermondii	374	Turkey	3.7	2.65	0.868	0.893
Meyerozyma guilliermondii	395	Germany	3.2	3.25	0.897	0.865
Meyerozyma guilliermondii	406	Turkey	3.5	3.2	0.88	0.867
Meyerozyma guilliermondii	427	Turkey	3.5	2.7	0.88	0.891
Meyerozyma guilliermondii	453	Russia	3.1	2.9	0.902	0.882
Meyerozyma guilliermondii	532	Brazil	3.5	1.3	0.88	0.95
Meyerozyma guilliermondii	620	Russia	3.4	3.1	0.886	0.872
Meyerozyma guilliermondii	659	UK	3.3	3.1	0.892	0.872
Meyerozyma guilliermondii	M4	UK	3.2	3.25	0.897	0.865
Millerozyma farinosa	356	Thailand	2.3	1.2	0.937	0.954
Nakaseomyces glabrata	670	UK	3.4	2.2	0.886	0.913
Nakazawaea holstii	610	Russia	3.35	2.5	0.889	0.9
Pichia fermentans	293	UK	2.1	1.3	0.944	0.95
Pichia fermentans	324	UK	2.25	1.2	0.938	0.954
Pichia kudriavzevii	294	UK	2.85	2.3	0.914	0.909
Pichia kudriavzevii	172	Netherlands	2.9	2.2	0.911	0.913
Pichia kudriavzevii	217	Netherlands	2.9	2.4	0.911	0.904
Pichia kudriavzevii	354	Thailand	3	2.05	0.907	0.919
Pichia kudriavzevii	380	Turkey	3.15	2.05	0.9	0.919
Pichia kudriavzevii	522	Brazil	2.9	3.8	0.911	0.836
Pichia kudriavzevii	641	UK	3	1.85	0.907	0.928
Pichia kudriavzevii	654	UK	3.15	2.35	0.9	0.907
Pichia manshurica	86	Netherlands	3	2.2	0.907	0.913
Pichia manshurica	120	UK	3	2.4	0.907	0.904
Pichia manshurica	123	UK	3	2.6	0.907	0.896
Pichia manshurica	124	UK	3	2.6	0.907	0.896
Pichia manshurica	169	Netherlands	2.65	2.5	0.923	0.9
Pichia manshurica	170	Netherlands	2.75	2.3	0.92	0.909
Pichia manshurica	171	Netherlands	2.7	2.2	0.921	0.913
Pichia manshurica	236	Thailand	2.8	2.6	0.916	0.896
Pichia manshurica	386	Turkey	2.75	2.35	0.92	0.907
Pichia manshurica	458	Russia	2.6	2.5	0.925	0.9
Pichia manshurica	478	Brazil	2.8	2.4	0.916	0.904
Pichia manshurica	521	Brazil	2.9	2.5	0.911	0.9
Pichia membranifaciens	173	UK	2.75	1.9	0.92	0.926
Pichia membranifaciens	210	Netherlands	2.9	3.2	0.911	0.867
Pichia occidentalis	237	Thailand	2.9	2	0.911	0.921
Pichia occidentalis	454	Russia	2.9	1.85	0.911	0.928
Pichia occidentalis	473	Brazil	3	1.8	0.907	0.93
Pichia occidentalis	523	Brazil	3	2.05	0.907	0.919
Pichia scutulata sister sp.	320	UK	2.65	1.4	0.923	0.946
Pichia sp.nov.	202	UK	2.5	1.5	0.928	0.942
Pseudozyma aphidis	554	Brazil	1.3	1.3	0.968	0.95
Pseudozyma sp.nov.	336	Thailand	2.6	1.5	0.925	0.942
Pseudozyma sp.nov.	658	UK	2.9	2.4	0.911	0.904
Rhodosporidium fluviale	379	Turkey	2.2	1.5	0.94	0.942

Yeast Species	Strain	Origin	MIC (M)		A_w	
			Glucose	NaCl	Glucose	NaCl
Rhodosporidium fluviale	426	Turkey	2.65	1.3	0.923	0.95
Rhodosporidium fluviale	491	Brazil	2.75	1.3	0.92	0.95
Rhodosporidium fluviale	549	Brazil	2.4	1.45	0.933	0.944
Rhodotorula colostri	623	Russia	2.7	1.3	0.921	0.95
Rhodotorula dairenensis	616	Russia	2.85	2.5	0.914	0.9
Rhodotorula glutinis	92	Israel	3	2.5	0.907	0.9
Rhodotorula glutinis	96	France	3	2.5	0.907	0.9
Rhodotorula glutinis	330	Thailand	2.2	1.95	0.94	0.924
Rhodotorula glutinis	412	Turkey	2.25	1.8	0.938	0.93
Rhodotorula glutinis sister sp.	NCYC 59		2.5	1.1	0.928	0.957
Rhodotorula glutinis sister sp.	93	France	2.5	1.9	0.928	0.926
Rhodotorula graminis	167	Thailand	2.5	2	0.928	0.921
Rhodotorula graminis	587	S Africa	2.2	1.75	0.94	0.933
Rhodotorula graminis	615	Russia	2.85	2	0.914	0.921
Rhodotorula minuta	378	Turkey	1.9	1.2	0.951	0.954
Rhodotorula mucilaginosa	90	UK	3.5	2.6	0.88	0.896
Rhodotorula mucilaginosa	95	UK	3	2.5	0.907	0.9
Rhodotorula mucilaginosa	143	Netherlands	3	2.6	0.907	0.896
Rhodotorula mucilaginosa	218	Netherlands	2.75	2.6	0.92	0.896
Rhodotorula mucilaginosa	222	Netherlands	2.7	2.9	0.921	0.882
Rhodotorula mucilaginosa	329	Thailand	2.6	2.62	0.925	0.895
Rhodotorula mucilaginosa	363	Turkey	2.9	2.45	0.911	0.902
Rhodotorula mucilaginosa	401	Turkey	3	2.55	0.907	0.898
Rhodotorula mucilaginosa	441	Turkey	2.9	2.55	0.911	0.898
Rhodotorula mucilaginosa	468	Russia	2.75	2.55	0.92	0.898
Rhodotorula mucilaginosa	469	Russia	3	2.45	0.907	0.902
Rhodotorula mucilaginosa	489	Brazil	2.95	2.45	0.909	0.902
Rhodotorula mucilaginosa	550	Brazil	3	2.55	0.907	0.898
Rhodotorula mucilaginosa	581	S Africa	2.5	2.2	0.928	0.913
Rhodotorula mucilaginosa	668	UK	2.5	2.2	0.928	0.913
Rhodotorula mucilaginosa	676	UK	2.75	2.1	0.92	0.917
Rhodotorula mucilaginosa	677	UK	2.5	2.2	0.928	0.913
Rhodotorula mucilaginosa	NCYC 195		3.5	2.5	0.88	0.9
Rhodotorula nothofagi	154	Netherlands	2.75	1.5	0.92	0.942
Rhodotorula nothofagi	602	Russia	2.75	1.6	0.92	0.939
Rhodotorula slooffiae	208	Netherlands	2.4	1.55	0.933	0.94
Rhodotorula slooffiae	585	S Africa	1.8	1.4	0.954	0.946
Rhodotorula sp.nov.	101	UK	3	2.2	0.907	0.913
Saccharomyces bayanus var *bayanus*	24	UK	3	1.8	0.907	0.93
Saccharomyces bayanus var *bayanus*	59	UK	3	2	0.907	0.921
Saccharomyces bayanus var *bayanus*	NCYC 2669		2.7	1.95	0.921	0.924
Saccharomyces bayanus var *uvarum*	97	UK	3	2	0.907	0.921
Saccharomyces bayanus var *uvarum*	25	UK	3	2.2	0.907	0.913
Saccharomyces bayanus var *uvarum*	117	UK	3	2.4	0.907	0.904
Saccharomyces cariocanus	NCYC 2890		3.1	2	0.902	0.921
Saccharomyces cerevisiae	22	UK	3.5	2	0.88	0.921
Saccharomyces cerevisiae	47	UK	3.5	2.1	0.88	0.917
Saccharomyces cerevisiae	48	UK	3.5	2.3	0.88	0.909
Saccharomyces cerevisiae	56	UK	3	2.2	0.907	0.913

Yeast Species	Strain	Origin	MIC (M)		A_w	
			Glucose	NaCl	Glucose	NaCl
Saccharomyces cerevisiae	62	UK	3.5	2	0.88	0.921
Saccharomyces cerevisiae	63	UK	3	1.9	0.907	0.926
Saccharomyces cerevisiae	64	UK	3	1.8	0.907	0.93
Saccharomyces cerevisiae	65	UK	3.2	2	0.897	0.921
Saccharomyces cerevisiae	125	France	3.5	2.4	0.88	0.904
Saccharomyces cerevisiae	174	UK	3.25	2.2	0.895	0.913
Saccharomyces cerevisiae	244	Netherlands	3.25	2.1	0.895	0.917
Saccharomyces cerevisiae	253	Netherlands	3.3	1.9	0.892	0.926
Saccharomyces cerevisiae	273	UK	3.2	2.2	0.897	0.913
Saccharomyces cerevisiae	282	Netherlands	3.25	1.7	0.895	0.935
Saccharomyces cerevisiae	291	UK	3.1	2.2	0.902	0.913
Saccharomyces cerevisiae	292	UK	3.25	2	0.895	0.921
Saccharomyces cerevisiae	308	Belgium	4.1	2.2	0.84	0.913
Saccharomyces cerevisiae	317	UK	3.25	1.8	0.895	0.93
Saccharomyces cerevisiae	359	Turkey	2.5	1.75	0.928	0.933
Saccharomyces cerevisiae	632	UK	3	1.8	0.907	0.93
Saccharomyces cerevisiae	633	UK	3	1.8	0.907	0.93
Saccharomyces cerevisiae	634	UK	3	1.8	0.907	0.93
Saccharomyces cerevisiae	635	UK	3	1.85	0.907	0.928
Saccharomyces cerevisiae	636	UK	3	1.8	0.907	0.93
Saccharomyces cerevisiae	656	UK	3.45	2.15	0.883	0.915
Saccharomyces cerevisiae	667	UK	2.75	1.85	0.92	0.928
Saccharomyces cerevisiae	BY4741	Euroscarf	3	2.3	0.907	0.909
Saccharomyces cerevisiae	BY4742	Euroscarf	3.2	2.2	0.897	0.913
Saccharomyces cerevisiae	BY4743	Euroscarf	3	2.15	0.907	0.915
Saccharomyces cerevisiae	NCYC 366		3.5	2	0.88	0.921
Saccharomyces cerevisiae	NCYC 87		3.2	2.4	0.897	0.904
Saccharomyces cerevisiae	X2180-1B		3.5	2.3	0.88	0.909
Saccharomyces kudriavzevii	NCYC 2889		2.8	1.5	0.916	0.942
Saccharomyces mikatae	NCYC 2888		3.1	2	0.902	0.921
Saccharomyces paradoxus	NCYC 2600		2.8	1.9	0.916	0.926
Saccharomyces paradoxus	NCYC 2601		3.15	1.95	0.9	0.924
Saccharomyces pastorianus	NCYC 392		2.75	1.75	0.92	0.933
Saccharomyces pastorianus	201	Netherlands	3.1	2.4	0.902	0.904
Saccharomycodes ludwigii	NCYC 3532		2.7	1.55	0.921	0.94
Saccharomycodes ludwigii	NCYC 730		2.3	1.45	0.937	0.944
Saccharomycodes ludwigii	NCYC 731		2.55	1.5	0.927	0.942
Saccharomycodes ludwigii	NCYC 732		2.25	1.5	0.938	0.942
Saccharomycodes ludwigii	NCYC 734		2.35	1.6	0.935	0.939
Saccharomycodes ludwigii	NCYC 849		2.35	1.55	0.935	0.94
Saturnispora sp.nov.	186	UK	2.5	1.3	0.928	0.95
Schizosaccharomyces pombe	NCYC 1346		4.25	1.1	0.828	0.957
Schizosaccharomyces pombe	NCYC 2722		3.5	0.6	0.88	0.975
Schwanniomyces etchelsii	213	Netherlands	2.7	1.6	0.921	0.939
Sporidiobolus johnsonii	498	Brazil	2.75	2.05	0.92	0.919
Sporidiobolus metaroseus	648	UK	2.7	1.85	0.921	0.928
Sporidiobolus metaroseus	649	UK	2.95	1.85	0.909	0.928
Sporidiobolus salmonicolor	221	Netherlands	2.65	2.1	0.923	0.917
Sporobolomyces sp.nov.	614	Russia	2.65	1.7	0.923	0.935

Yeast Species	Strain	Origin	MIC (M)		A_w	
			Glucose	NaCl	Glucose	NaCl
Torulaspora delbrueckii	137	UK	3.5	2.6	0.88	0.896
Torulaspora delbrueckii	207	Netherlands	3.5	2.75	0.88	0.889
Torulaspora delbrueckii	366	Turkey	3.5	2.7	0.88	0.891
Torulaspora delbrueckii	410	Turkey	3.6	2.7	0.874	0.891
Torulaspora delbrueckii	456	Russia	3.4	2.8	0.886	0.886
Torulaspora delbrueckii	529	Brazil	3.9	2.5	0.854	0.9
Torulaspora delbrueckii	624	Russia	3.7	2.8	0.868	0.886
Torulaspora delbrueckii	631	UK	3.9	2.55	0.854	0.898
Torulaspora delbrueckii	638	UK	3.65	2.55	0.871	0.898
Torulaspora delbrueckii	653	UK	3.7	2.6	0.868	0.896
Torulaspora globosa	NCYC 820		2.75	1.55	0.92	0.94
Torulaspora microellipsoides	NCYC 2568		3	0.8	0.907	0.968
Torulaspora microellipsoides	M11	UK	3	0.8	0.907	0.968
Torulaspora microellipsoides	NCYC 411		3.05	0.65	0.904	0.973
Torulaspora pretoriensis	NCYC 524		3.7	1.4	0.868	0.946
Trichosporon asahii	331	Thailand	2.2	2.3	0.94	0.909
Trichosporon asahii	429	Turkey	2.6	2.3	0.925	0.909
Trichosporon asahii	488	Brazil	2.6	2.1	0.925	0.917
Trichosporon asahii	540	Brazil	2.2	1.9	0.94	0.926
Trichosporon coremiiforme	459	Russia	2.35	2.1	0.935	0.917
Trichosporon coremiiforme	650	UK	2.1	2.45	0.944	0.902
Trichosporon coremiiforme	669	UK	2.3	2.2	0.937	0.913
Trichosporon coremiiforme	678	UK	2.4	2.05	0.933	0.919
Trichosporon domesticum	651	UK	2.65	1.4	0.923	0.946
Trichosporon gracile	647	UK	1.7	1.4	0.957	0.946
Trichosporon jirovecii	405	Turkey	2.05	1	0.946	0.96
Trichosporon jirovecii	542	Brazil	2.1	0.85	0.944	0.966
Trichosporon jirovecii	652	UK	1.8	1.35	0.954	0.948
Trichosporon mucoides	381	Turkey	2.9	2.35	0.911	0.907
Trichosporon mucoides	466	Russia	2.5	2.1	0.928	0.917
Trichosporon mycotoxinivorans	541	Brazil	2.9	1.6	0.911	0.939
Trichosporon ovoides	409	Turkey	2.8	1.75	0.916	0.933
Trichosporon sp.nov.	339	Thailand	2.65	1.5	0.923	0.942
Wickerhamomyces anomalus	54	France	3.5	3	0.88	0.876
Wickerhamomyces anomalus	70	Israel	3.5	2.8	0.88	0.886
Wickerhamomyces anomalus	71	France	3.5	2.8	0.88	0.886
Wickerhamomyces anomalus	73	France	3.5	3.2	0.88	0.867
Wickerhamomyces anomalus	88	UK	3.5	2.8	0.88	0.886
Wickerhamomyces anomalus	156	Netherlands	3.8	2.9	0.861	0.882
Wickerhamomyces anomalus	364	Turkey	3.4	2.07	0.886	0.918
Wickerhamomyces anomalus	415	Turkey	3.75	2.7	0.864	0.891
Wickerhamomyces anomalus	447	Turkey	3.65	2.7	0.871	0.891
Wickerhamomyces anomalus	455	Russia	3.7	2.65	0.868	0.893
Wickerhamomyces anomalus	482	Brazil	3.85	2.3	0.858	0.909
Wickerhamomyces anomalus	516	Brazil	4	1.7	0.849	0.935
Wickerhamomyces anomalus	582	S Africa	3.7	2.65	0.868	0.893
Wickerhamomyces anomalus	612	Russia	3.6	2.95	0.874	0.879
Wickerhamomyces anomalus	613	Russia	3.65	2.95	0.871	0.879
Wickerhamomyces anomalus	NCYC 18		3	2	0.907	0.921

Yeast Species	Strain	Origin	MIC (M)		A_w	
			Glucose	NaCl	Glucose	NaCl
Wickerhamomyces anomalus	NCYC 711		3.5	2.8	0.88	0.886
Wickerhamomyces subpelliculosus	IFFI 01014		3.2	3.65	0.897	0.845
Yarrowia lipolytica	149	Netherlands	2.9	2.3	0.911	0.909
Yarrowia lipolytica	205	Netherlands	2.8	2.4	0.916	0.904
Yarrowia lipolytica	472	Brazil	2.85	2.6	0.914	0.896
Yarrowia lipolytica	474	Brazil	2.8	2.65	0.916	0.893
Yarrowia lipolytica	499	Brazil	3.15	2.4	0.9	0.904
Yarrowia lipolytica	500	Brazil	3.05	2.45	0.904	0.902
Yarrowia lipolytica	560	Brazil	2.8	2.5	0.916	0.9
Yarrowia lipolytica	619	Russia	3	2.5	0.907	0.9
Yarrowia lipolytica	642	UK	2.55	2.5	0.927	0.9
Yarrowia lipolytica	661	UK	2.9	2.2	0.911	0.913
Zygoascus hellenicus	533	Brazil	3.4	1.65	0.886	0.937
Zygosaccharomyces bailii	2	UK	3.85	2.4	0.858	0.904
Zygosaccharomyces bailii	4	USA	4.05	2.6	0.844	0.896
Zygosaccharomyces bailii	5	USA	4	3.2	0.849	0.867
Zygosaccharomyces bailii	6	USA	3.95	3.2	0.851	0.867
Zygosaccharomyces bailii	7	USA	3.95	2.6	0.851	0.896
Zygosaccharomyces bailii	8	USA	3.9	2.4	0.854	0.904
Zygosaccharomyces bailii	9	USA	3.95	2.6	0.851	0.896
Zygosaccharomyces bailii	10	USA	3.85	2.4	0.858	0.904
Zygosaccharomyces bailii	11	USA	3.9	2.6	0.854	0.896
Zygosaccharomyces bailii	12	USA	3.95	2.6	0.851	0.896
Zygosaccharomyces bailii	13	USA	4	3	0.849	0.876
Zygosaccharomyces bailii	15	Netherlands	3.9	2.8	0.854	0.886
Zygosaccharomyces bailii	16	Netherlands	4	2.6	0.849	0.896
Zygosaccharomyces bailii	17	UK	4	2.6	0.849	0.896
Zygosaccharomyces bailii	18	UK	3.5	2.6	0.88	0.896
Zygosaccharomyces bailii	19	UK	3.9	3	0.854	0.876
Zygosaccharomyces bailii	20	UK	3.8	2.6	0.861	0.896
Zygosaccharomyces bailii	21	UK	3.5	2.5	0.88	0.9
Zygosaccharomyces bailii	52	Netherlands	4	3	0.849	0.876
Zygosaccharomyces bailii	80	Mexico	4.1	3.4	0.84	0.858
Zygosaccharomyces bailii	105	UK	4	2.8	0.849	0.886
Zygosaccharomyces bailii	106	UK	4.05	2.9	0.844	0.882
Zygosaccharomyces bailii	107	UK	3.95	2.9	0.851	0.882
Zygosaccharomyces bailii	108	UK	4	2.9	0.849	0.882
Zygosaccharomyces bailii	112	Belgium	3.9	2.4	0.854	0.904
Zygosaccharomyces bailii	114	Belgium	4.25	2.4	0.828	0.904
Zygosaccharomyces bailii	119	Netherlands	4	2.8	0.849	0.886
Zygosaccharomyces bailii	194	USA	4.1	3	0.84	0.876
Zygosaccharomyces bailii	280	S Africa	3.9	3.05	0.854	0.874
Zygosaccharomyces bailii	362	Turkey	4.05	2.5	0.844	0.9
Zygosaccharomyces bailii	475	Brazil	4.1	3	0.84	0.876
Zygosaccharomyces bailii	503	UK	4.2	1.95	0.832	0.924
Zygosaccharomyces bailii	505	UK	4.25	1.6	0.828	0.939
Zygosaccharomyces bailii	592	Phillipines	4.25	3.1	0.828	0.872
Zygosaccharomyces bailii	593	Phillipines	4	2.9	0.849	0.882
Zygosaccharomyces bailii	594	Sweden	3.2	2.3	0.897	0.909

Yeast Species	Strain	Origin	MIC (M)		A_w	
			Glucose	NaCl	Glucose	NaCl
Zygosaccharomyces bailii	595	Spain	4.05	2.85	0.844	0.884
Zygosaccharomyces bailii	M10	UK	3.9	2.3	0.854	0.909
Zygosaccharomyces bailii	M5	UK	4.25	3.1	0.828	0.872
Zygosaccharomyces bailii	M6	UK	4	2.9	0.849	0.882
Zygosaccharomyces bailii	M7	UK	4.05	2.85	0.844	0.884
Zygosaccharomyces bailii	M8	UK	4	2.83	0.849	0.885
Zygosaccharomyces bailii	NCYC 1416		3.5	2.2	0.88	0.913
Zygosaccharomyces bailii	NCYC 1766		4	2.6	0.849	0.896
Zygosaccharomyces bisporus	28	Israel	4.35	3.2	0.821	0.867
Zygosaccharomyces bisporus	104	UK	4	2.8	0.849	0.886
Zygosaccharomyces bisporus	133	UK	4	3	0.849	0.876
Zygosaccharomyces bisporus	134	UK	4.5	3.2	0.807	0.867
Zygosaccharomyces bisporus	257	Netherlands	3.9	2.85	0.854	0.884
Zygosaccharomyces bisporus	367	Turkey	4.15	2.48	0.836	0.901
Zygosaccharomyces bisporus	390	Turkey	4.25	2.35	0.828	0.907
Zygosaccharomyces bisporus	391	Turkey	4.3	2.4	0.825	0.904
Zygosaccharomyces bisporus	494	Brazil	4.5	2.7	0.807	0.891
Zygosaccharomyces bisporus	NCYC 1495		4	3.2	0.849	0.867
Zygosaccharomyces bisporus	NCYC 171		4	2.8	0.849	0.886
Zygosaccharomyces bisporus	NRRL 1228		3.25	2.5	0.895	0.9
Zygosaccharomyces bisporus	NRRL12627		3.25	2.6	0.895	0.896
Zygosaccharomyces bisporus	NRRL 7253		4.5	3.05	0.807	0.874
Zygosaccharomyces bisporus	NRRL 7684		3.75	2.6	0.864	0.896
Zygosaccharomyces kombuchaensis	198	USA	3.1	2.1	0.902	0.917
Zygosaccharomyces kombuchaensis	199	USA	2.9	2	0.911	0.921
Zygosaccharomyces kombuchaensis	200	USA	3.25	2.05	0.895	0.919
Zygosaccharomyces kombuchaensis	NCYC 2969		3.5	2	0.88	0.921
Zygosaccharomyces lentus	36	UK	3.5	2	0.88	0.921
Zygosaccharomyces lentus	37	UK	3.5	2	0.88	0.921
Zygosaccharomyces lentus	38	UK	4	2.6	0.849	0.896
Zygosaccharomyces lentus	39	France	3.5	2.6	0.88	0.896
Zygosaccharomyces lentus	40	UK	3.5	2.2	0.88	0.913
Zygosaccharomyces lentus	103	UK	3.5	2.4	0.88	0.904
Zygosaccharomyces lentus	398	UK	3.75	2.6	0.864	0.896
Zygosaccharomyces lentus	M9	UK	3.75	2.6	0.864	0.896
Zygosaccharomyces lentus	TNO 0566		3.8	2.7	0.861	0.891
Zygosaccharomyces lentus	TNO 0567		3.7	2.2	0.868	0.913
Zygosaccharomyces lentus	TNO 0569		3.9	2.3	0.854	0.909
Zygosaccharomyces lentus	TNO 0572		3.75	2.4	0.864	0.904
Zygosaccharomyces mellis	139	UK	5	3.4	0.756	0.858
Zygosaccharomyces mellis	141	UK	5	3	0.756	0.876
Zygosaccharomyces mellis	142	UK	5	3	0.756	0.876
Zygosaccharomyces mellis	192	USA	4.6	3.3	0.8	0.862
Zygosaccharomyces mellis	193	USA	4.7	3.4	0.79	0.858
Zygosaccharomyces mellis	195	USA	4.8	3.1	0.78	0.872
Zygosaccharomyces mellis	196	USA	4.75	3.2	0.785	0.867
Zygosaccharomyces mellis	197	USA	4.9	3.5	0.769	0.852
Zygosaccharomyces mellis	NCYC 2403		5	2.2	0.756	0.913
Zygosaccharomyces rouxii	33	UK	5	3.6	0.756	0.847

Yeast Species	Strain	Origin	MIC (M)		A_w	
			Glucose	NaCl	Glucose	NaCl
Zygosaccharomyces rouxii	34	UK	5.5	3.8	0.682	0.836
Zygosaccharomyces rouxii	35	UK	5.5	3.4	0.682	0.858
Zygosaccharomyces rouxii	115	UK	5.5	3.6	0.682	0.847
Zygosaccharomyces rouxii	116	UK	5.5	3.4	0.682	0.858
Zygosaccharomyces rouxii	140	Germany	4.5	3	0.807	0.876
Zygosaccharomyces rouxii	231	Denmark	4.8	3.6	0.78	0.847
Zygosaccharomyces rouxii	232	Denmark	4.9	3.75	0.769	0.839
Zygosaccharomyces rouxii	233	Denmark	5	3.7	0.756	0.842
Zygosaccharomyces rouxii	239	Netherlands	5.4	3.7	0.7	0.842
Zygosaccharomyces rouxii	241	Netherlands	4.75	3.9	0.785	0.832
Zygosaccharomyces rouxii	254	Netherlands	4.7	3.2	0.79	0.867
Zygosaccharomyces rouxii	255	Netherlands	4.75	3.3	0.785	0.862
Zygosaccharomyces rouxii	265	Netherlands	3.55	2.4	0.877	0.904
Zygosaccharomyces rouxii	596	Spain	5.25	3.3	0.724	0.862
Zygosaccharomyces rouxii	597	Spain	5.25	3.4	0.724	0.858
Zygosaccharomyces rouxii	ATCC66069		4.8	3.1	0.78	0.872
Zygosaccharomyces rouxii	CBS 4021		4.25	3.25	0.828	0.865
Zygosaccharomyces rouxii	CBS 4837		4.75	3.5	0.785	0.852
Zygosaccharomyces rouxii	CBS 681		5	3.7	0.756	0.842
Zygosaccharomyces rouxii	IFFI 01378		3.5	2.9	0.88	0.882
Zygosaccharomyces rouxii	IFFI 01417		3.9	3.2	0.854	0.867
Zygosaccharomyces rouxii	IFFI 01708		5.3	3.8	0.717	0.836
Zygosaccharomyces rouxii	M2	UK	5.3	3.4	0.717	0.858
Zygosaccharomyces rouxii	M3	UK	5.1	3.3	0.745	0.862
Zygosaccharomyces rouxii	NCYC 381		5	3.6	0.756	0.847
Zygosaccharomyces rouxii	NCYC 568		5	3.4	0.756	0.858
Zygosaccharomyces rouxii	NCYC 579		5	3.6	0.756	0.847
Zygosaccharomyces rouxii	NRRL 2547		5.15	3.8	0.738	0.836
Zygosaccharomyces rouxii hybrid	252	Netherlands	4	3.2	0.849	0.867
Zygosaccharomyces rouxii hybrid	258	Netherlands	3.25	2.95	0.895	0.879
Zygosaccharomyces rouxii hybrid	259	Netherlands	3.75	2.9	0.864	0.882
Zygosaccharomyces rouxii hybrid	266	Netherlands	3.85	3.35	0.858	0.86
Zygosaccharomyces rouxii hybrid	ATCC13356		4.85	3.9	0.775	0.832
Zygosaccharomyces rouxii hybrid	ATCC46261		4.65	3.8	0.795	0.836
Zygosaccharomyces rouxii hybrid	IFFI 01379		4	3.3	0.849	0.862
Zygosaccharomyces rouxii hybrid	IFFI 01711		4.7	3.75	0.79	0.839
Zygosaccharomyces rouxii hybrid	IFFI 01712		5.05	3.45	0.752	0.855
Zygosaccharomyces rouxii hybrid	NCYC 3363		3.75	2.4	0.864	0.904
Zygosaccharomyces rouxii hybrid	NRRL 2547		4.25	3.25	0.828	0.865
Zygosaccharomyces sp.nov.	IFFI 01710		4.65	3.6	0.795	0.847
Zygosaccharomyces sp.nov.	IFFI 01709		4.85	3.4	0.775	0.858
Zygosaccharomyces sp.nov.	NCYC 3265		4.9	3.65	0.769	0.845
Zygotorulaspora florentina	NCYC 2513		3.5	1.6	0.88	0.939
Zygotorulaspora florentina	44	UK	3.6	1.5	0.874	0.942
Zygotorulaspora mrakii	NCYC 2489		3	1.9	0.907	0.926
		**Mean**	**3.208**	**2.316**	**0.889**	**0.907**
