# Supplementary material for: Extreme Osmotolerance and Halotolerance in Food-Relevant Yeasts and the Role of Glycerol-Dependent Cell Individuality
Source: Front Microbiol. 2019 Jan 9;9:3238. doi: 10.3389/fmicb.2018.03238 (PMC6333755; doi:10.3389/fmicb.2018.03238)
Supplement: Supplementary file 2 [file Data_Sheet_2.PDF]

Table S2 Comparison of the glycerol and trehalose contents of wild-type cells of *S. cerevisiae* BY4741, versus deletion mutants *gpd1Δ* and *tps1Δ*. Glycerol content was measured in cultures grown for 2 weeks in 1.4 M, 0.8 M, or 0 M salt in YEPD. Trehalose content was measured in stationary-phase cultures after 2 days growth, as used for inoculation. Data are the means and SDs of duplicate cultures.

Glycerol content pMoles/cell

	**WT**		***Δgpd1***		***Δtps1***	
	Mean	SD	Mean	SD	Mean	SD
1.4M salt	35.99	27.47				
0.8M salt	6.44	0.97	3.32	0.08	5.87	1.57
0 salt	1.57	0.17	1.51	0.19	1.79	0.07

Trehalose content pgrams/cell

**WT**		***gpd1Δ***		***tps1Δ***	
Mean	SD	Mean	SD	Mean	SD
4.315	0.004	4.951	0.171	0.469	0.016

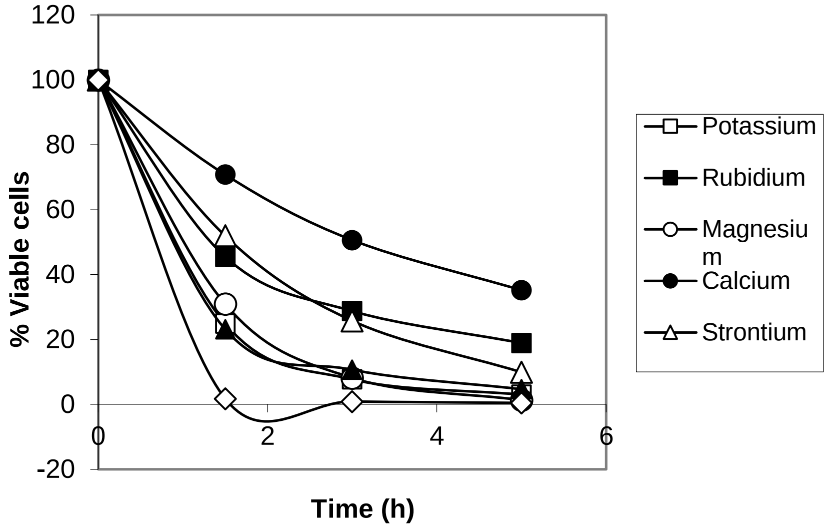

FIGURE S1 Comparison of cell death of *Saccharomyces cerevisiae* BY4741 caused by chloride salts and polyols. Death curves following inoculation into YEPD containing the MIC levels of chlorides, glycerol or sorbitol. Samples were taken over time and plated out onto YEPD agar. Samples are shown of potassium chloride (2.225 M open squares), rubidium chloride (1.85 M closed squares), magnesium chloride (1.4 M open circles), calcium chloride (1.05 M closed circles), strontium chloride (1.25M open triangles), glycerol (3.8 M closed triangles) and sorbitol (2.86 M open diamonds).

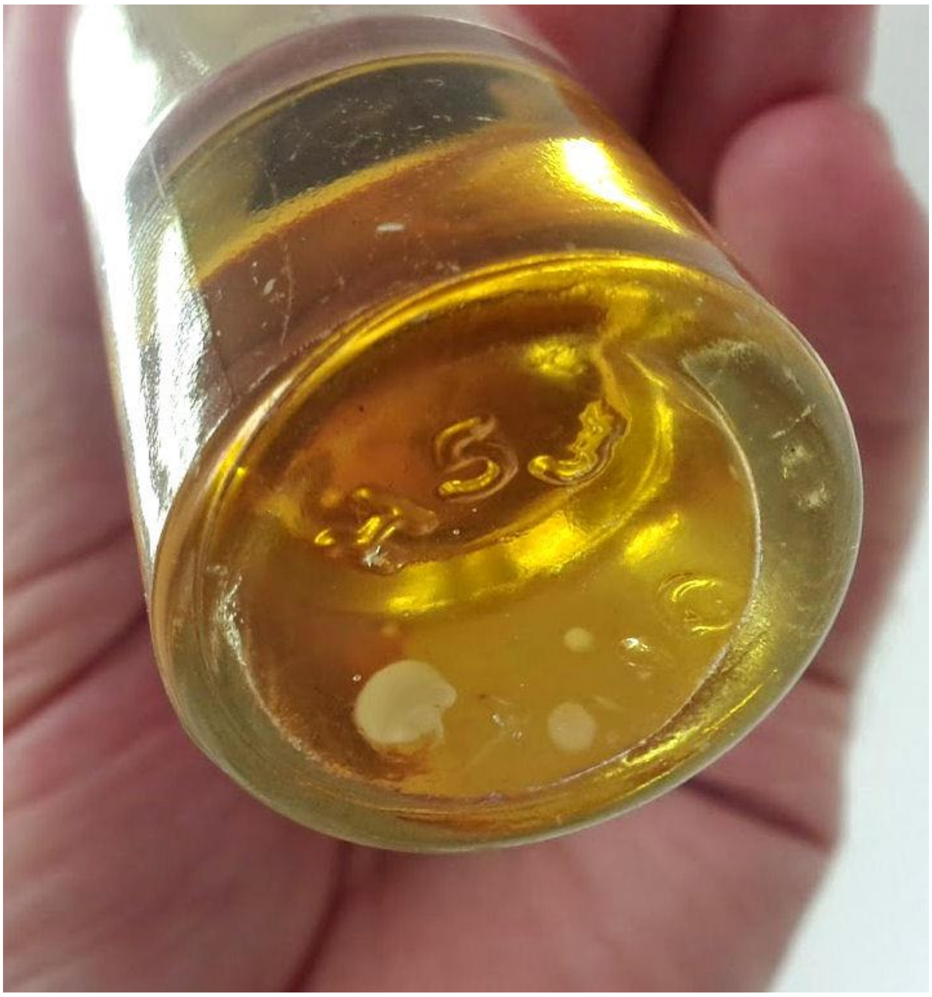

FIGURE S2 Small numbers of yeast colonies (*Saccharomyces cerevisiae* BY4741) growing in 2 M salt (NaCl) in 10 mls YEPD pH 4.0, from an inoculum of 10^4 cells.

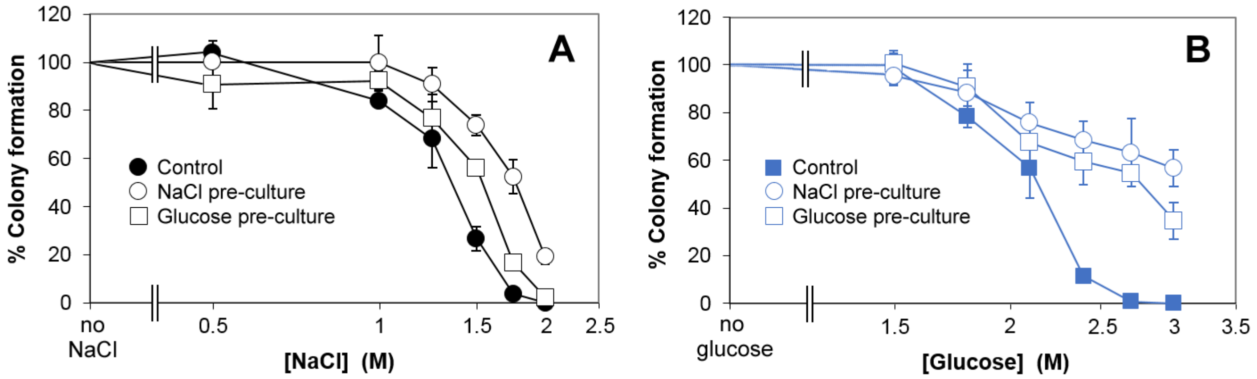

FIGURE S3 Pre-culture with low salt or glucose increases heteroresistance to chronic-glucose but not -salt stress. *S. cerevisiae* BY4741 was pre-cultured for 3 weeks in standard YEPD broth cultures (control), 2 M NaCl, or 2.7 M glucose, then plated to YEPD agar supplemented with NaCl salt **(A)** or glucose **(B)**. Colonies were enumerated after incubation for 28 days at 25°C. Data are means ± SD from triplicate cultures.
